# An Introductory Review of Deep Learning for Prediction Models With Big Data

**DOI:** 10.3389/frai.2020.00004

**Published:** 2020-02-28

**Authors:** Frank Emmert-Streib, Zhen Yang, Han Feng, Shailesh Tripathi, Matthias Dehmer

**Affiliations:** ^1^Predictive Society and Data Analytics Lab, Faculty of Information Technology and Communication Sciences, Tampere University, Tampere, Finland; ^2^Institute of Biosciences and Medical Technology, Tampere, Finland; ^3^School of Management, University of Applied Sciences Upper Austria, Steyr, Austria; ^4^Department of Biomedical Computer Science and Mechatronics, University for Health Sciences, Medical Informatics and Technology (UMIT), Hall in Tyrol, Austria; ^5^College of Artificial Intelligence, Nankai University, Tianjin, China

**Keywords:** deep learning, artificial intelligence, machine learning, neural networks, prediction models, data science

## Abstract

Deep learning models stand for a new learning paradigm in artificial intelligence (AI) and machine learning. Recent breakthrough results in image analysis and speech recognition have generated a massive interest in this field because also applications in many other domains providing big data seem possible. On a downside, the mathematical and computational methodology underlying deep learning models is very challenging, especially for interdisciplinary scientists. For this reason, we present in this paper an introductory review of deep learning approaches including Deep Feedforward Neural Networks (D-FFNN), Convolutional Neural Networks (CNNs), Deep Belief Networks (DBNs), Autoencoders (AEs), and Long Short-Term Memory (LSTM) networks. These models form the major core architectures of deep learning models currently used and should belong in any data scientist's toolbox. Importantly, those core architectural building blocks can be composed flexibly—in an almost Lego-like manner—to build new application-specific network architectures. Hence, a basic understanding of these network architectures is important to be prepared for future developments in AI.

## 1. Introduction

We are living in the big data era where all areas of science and industry generate massive amounts of data. This confronts us with unprecedented challenges regarding their analysis and interpretation. For this reason, there is an urgent need for novel machine learning and artificial intelligence methods that can help in utilizing these data. Deep learning (DL) is such a novel methodology currently receiving much attention (Hinton et al., 2006). DL describes a family of learning algorithms rather than a single method that can be used to learn complex prediction models, e.g., multi-layer neural networks with many hidden units (LeCun et al., 2015). Importantly, deep learning has been successfully applied to several application problems. For instance, a deep learning method set the record for the classification of handwritten digits of the MNIST data set with an error rate of 0.21% (Wan et al., 2013). Further application areas include image recognition (Krizhevsky et al., 2012a; LeCun et al., 2015), speech recognition (Graves et al., 2013), natural language understanding (Sarikaya et al., 2014), acoustic modeling (Mohamed et al., 2011) and computational biology (Leung et al., 2014; Alipanahi et al., 2015; Zhang S. et al., 2015; Smolander et al., 2019a,b).

Models of artificial neural networks have been used since about the 1950s (Rosenblatt, 1957); however, the current wave of deep learning neural networks started around 2006 (Hinton et al., 2006). A common characteristic of the many variations of supervised and unsupervised deep learning models is that these models have many layers of hidden neurons learned, e.g., by a Restricted Boltzmann Machine (RBM) in combination with Backpropagation and error gradients of the Stochastic Gradient Descent (Riedmiller and Braun, 1993). Due to the heterogeneity of deep learning approaches a comprehensive discussion is very challenging, and for this reason, previous reviews aimed at dedicated sub-topics. For instance, a bird's eye view without detailed explanations can be found in LeCun et al. (2015), a historic summary with many detailed references in Schmidhuber (2015) and reviews about application domains, e.g., image analysis (Rawat and Wang, 2017; Shen et al., 2017), speech recognition (Yu and Li, 2017), natural language processing (Young et al., 2018), and biomedicine (Cao et al., 2018).

In contrast, our review aims at an intermediate level, providing also technical details usually omitted. Given the interdisciplinary interest in deep learning, which is part of data science (Emmert-Streib and Dehmer, 2019a), this makes it easier for people new to the field to get started. The topics we selected are focused on the core methodology of deep learning approaches including Deep Feedforward Neural Networks (D-FFNN), Convolutional Neural Networks (CNNs), Deep Belief Networks (DBNs), Autoencoders (AEs), and Long Short-Term Memory (LSTM) networks. Further network architectures which we discuss help in understanding these core approaches.

This paper is organized as follows. In the section 2, we provide a historical overview of general developments of neural networks. Then in section 3, we discuss major architectures distinguishing neural networks. Thereafter, we discuss Deep Feedforward Neural Networks (section 4), Convolutional Neural Networks (section 5), Deep Belief Networks (section 6), Autoencoders (section 7) and Long Short-Term Memory networks (section 8) in detail. In section 9, we provide a discussion of important issues when learning neural network models. Finally, this paper finishes in section 10 with conclusions.

## 2. Key Developments of Neural Networks: A Time Line

The history of neural networks is long, and many people have contributed toward their development over the decades. Given the recent explosion of interest in deep learning, it is not surprising that the assignment of credit for key developments is not uncontroversial. In the following, we were aiming at an unbiased presentation highlighting only the most distinguished contributions.

In 1943, the first mathematical model of a neuron was created by McCulloch and Pitts (1943). This model aimed at providing an abstract formulation for the functioning of a neuron without mimicking the biophysical mechanism of a real biological neuron. It is interesting to note that this model did not consider learning.

In 1949, the first idea about biologically motivated learning in neural networks was introduced by Hebb (1949). Hebbian learning is a form of unsupervised learning of neural networks.

In 1957, the Perceptron was introduced by Rosenblatt (1957). The Perceptron is a single-layer neural network serving as a linear binary classifier. In the modern language of ANNs, a Perceptron uses the Heaviside function as an activation function (see Table 1).

**Table 1:** An overview of frequently used activation functions for neuron models.

**Activation function**	***ϕ*(x)**	***ϕ*^′^(x)**	**Values**
Hyperbolic tangent	$\tanh(x)=\frac{e^{x}-e^{-x}}{e^{x}+e^{-x}}$	1 − *ϕ*(*x*)^2^	(−1, 1)
Sigmoid	$S\left(x\right)=\frac{1}{1+e^{-x}}$	*ϕ*(*x*)(1 − *ϕ*(*x*))	(0, 1)
ReLu	$R(x)=\{\begin{array}{ll} 0 & \text{for }x<0\\ x & \text{for }x\geq 0 \end{array}$	$\{\begin{array}{ll} 0 & \text{for }x<0\\ 1 & \text{for }x\geq 0 \end{array}$	[0, ∞)
Heaviside function	$H(x)=\{\begin{array}{ll} 0 & \text{for }x<0\\ 1 & \text{for }x\geq 0 \end{array}$	*δ(x*)	[0, 1]
Signum function	$sgn(x)=\{\begin{array}{ll} -1 & \text{for }x<0\\ 0 & \text{for }x=0\\ 1 & \text{for }x>0 \end{array}$	*2δ(x*)	[−1, 1]
Softmax	$y_{i}=\frac{e^{x_{i}}}{\overset{n}{\underset{j}{\sum }}e^{x_{j}}}$	$\frac{\partial y_{i}}{\partial j}=y_{i}\delta_{ij}-y_{j}$	(0, 1)

In 1960, the Delta Learning rule for learning a Perceptron was introduced by Widrow and Hoff (1960). The Delta Learning rule, also known as Widrow & Hoff Learning rule or the Least Mean Square rule, is a gradient descent learning rule for updating the weights of the neurons. It is a special case of the backpropagation algorithm.

In 1968, a method called *Group Method of Data Handling* (GMDH) for training neural networks was introduced by Ivakhnenko (1968). These networks are widely considered the first deep learning networks of the *Feedforward Multilayer Perceptron* type. For instance, the paper (Ivakhnenko, 1971) used a deep GMDH network with 8 layers. Interestingly, the numbers of layers and units per layer could be learned and were not fixed from the beginning.

In 1969, an important paper by Minsky and Papert (1969) was published which showed that the XOR problem cannot be learned by a Perceptron because it is not linearly separable. This triggered a pause phase for neural networks called the “AI winter.”

In 1974, error backpropagation (BP) has been suggested to use in neural networks (Werbos, 1974) for learning the weighted in a supervised manner and applied in Werbos (1981). However, the method itself is older (see e.g., Linnainmaa, 1976).

In 1980, a hierarchical multilayered neural network for visual pattern recognition called *Neocognitron* was introduced by Fukushima (1980). After the deep GMDH networks (see above), the *Neocognitron* is considered the second artificial NN that deserved the attribute *deep*. It introduced *convolutional NNs* (today called CNNs). The Neocognitron is very similar to the architecture of modern, *supervised*, deep Feedforward Neural Networks (D-FFNN) (Fukushima, 2013).

In 1982, Hopfield introduced a content-addressable memory neural network, nowadays called Hopfield Network (Hopfield, 1982). Hopfield Networks are an example for recurrent neural networks.

In 1986, backpropagation reappeared in a paper by Rumelhart et al. (1986). They showed experimentally that this learning algorithm can generate useful internal representations and, hence, be of use for general neural network learning tasks.

In 1987, Terry Sejnowski introduced the NETtalk algorithm (Sejnowski and Rosenberg, 1987). The program learned how to pronounce English words and was able to improve over time.

In 1989, a Convolutional Neural Network was trained with the backpropagation algorithm to learn handwritten digits (LeCun et al., 1989). A similar system was later used to read handwritten checks and zip codes, processing cashed checks in the United States in the late 90s and early 2000s.

Note: In the 1980s, the second wave of neural network research emerged in great part via a movement called *connectionism* (Fodor and Pylyshyn, 1988). This wave lasted until the mid 1990s.

In 1991, Hochreiter studied a fundamental problem of any deep learning network, which relates to the problem of not being trainable with the backpropagation algorithm (Hochreiter, 1991). His study revealed that the signal propagated by backpropagation either decreases or increases without bounds. In case of a decay, this is proportional to the depth of the network. This is now known as the vanishing or exploding gradient problem.

In 1992, a first partial remedy to this problem has been suggested by Schmidhuber (1992). The idea was to pre-train a RNN in an unsupervised way to accelerate subsequent supervised learning. The studied network had more than 1,000 layers in the recurrent neural network.

In 1995, oscillatory neural networks have been introduced in Wang and Terman (1995). They have been used in various applications like image and speech segmentation and generating complex time series (Wang and Terman, 1997; Hoppensteadt and Izhikevich, 1999; Wang and Brown, 1999; Soman et al., 2018).

In 1997, the first supervised model for learning RNN was introduced by Hochreiter and Schmidhuber (1997), which was called Long Short-Term Memory (LSTM). A LSTM prevents the decaying error signal problem between layers by making the LSTM networks “remember” information for a longer period of time.

In 1998, the Stochastic Gradient Descent algorithm (gradient-based learning) was combined with the backpropagation algorithm for improving learning in CNN (LeCun et al., 1989). As a result, LeNet-5, a 7-level convolutional network, was introduced for classifying hand-written numbers on checks.

In 2006, is widely considered a breakthrough year because in Hinton et al. (2006) it was shown that neural networks called Deep Belief Networks can be efficiently trained by using a strategy called greedy layer-wise pre-training. This initiated the third wave of neural networks that made also the use of the term *deep learning* popular.

In 2012, Alex Krizhevsky won the ImageNet Large Scale Visual Recognition Challenge by using AlexNet, a Convolutional Neural Network utilizing a GPU and improved upon LeNet5 (see above) (LeCun et al., 1989). This success started a convolutional neural network renaissance in the deep learning community (see Neocognitron).

In 2014, generative adversarial networks were introduced in Goodfellow et al. (2014). The idea is that two neural networks compete with each other in a game-like manner. Overall, this establishes a generative model that can produce new data. This has been called “the coolest idea in machine learning in the last 20 years” by Yann LeCun.

In 2019, Yoshua Bengio, Geoffrey Hinton, and Yann LeCun were awarded the Turing Award for conceptual and engineering breakthroughs that have made deep neural networks a critical component of computing.

The reader interested in a more detailed early history of neural networks is referred to Schmidhuber (2015).

**Figure 1:**
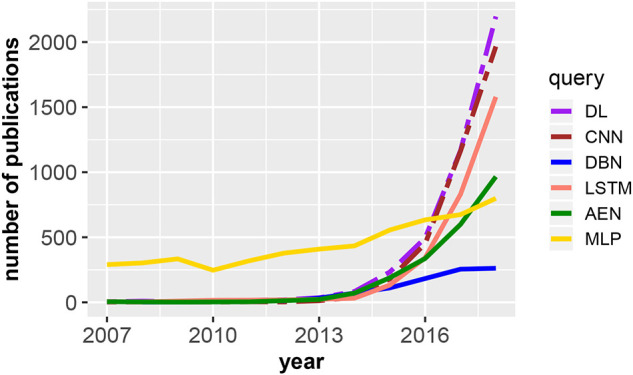
Number of publications in dependence on the publication year for DL, deep learning; CNN, convolutional neural network; DBN, deep belief network; LSTM, long short-term memory; AEN, autoencoder; and MLP, multilayer perceptron. The legend shows the search terms used to query the Web of Science publication database. The two dashed lines are scaled by a factor of 5 (deep learning) and 3 (convolutional neural network).

In Figure 1, we show the evolution of publications related to deep learning from the Web of Science publication database. Specifically, the figure shows the number of publications in dependence on the publication year for DL, deep learning; CNN, convolutional neural network; DBN, deep belief network; LSTM, long short-term memory; AEN, autoencoder; and MLP, multilayer perceptron. The two dashed lines are scaled by a factor of 5 (deep learning) and 3 (convolutional neural network), i.e., overall, for deep learning we found the majority of publications (in total 30, 230). Interestingly, most of these are in computer science (52.1%) and engineering (41.5%). In application areas, medical imaging (6.2%), robotics (2.6%), and computational biology (2.5%) received most attention. These observations are a reflection of the brief history of deep learning indicating that the methods are still under development.

In the following sections, we will discuss all of these methods in more detail because they represent the core methodology of deep learning. In addition, we present background information about general artificial neural networks as far as this is needed for a better understanding of the DL methods.

## 3. Architectures of Neural Networks

Artificial Neural Networks (ANNs) are mathematical models that have been motivated by the functioning of the brain. However, the models we discuss in the following do not aim at providing biologically realistic models. Instead, the purpose of these models is to analyze data.

### 3.1. Model of an Artificial Neuron

**Figure 2:**
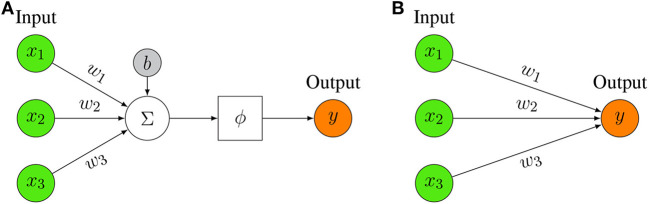
**(A)** Representation of a mathematical artificial neuron model. The input to the neuron is summed up and filtered by activation function ϕ (for examples see Table 1). **(B)** Simplified Representation of an artificial neuron model. Only the key elements are depicted, i.e., the input, the output, and the weights.

The basic entity of any neural network is a model of a neuron. In Figure 2A, we show such a model of an artificial neuron.

The basic idea of a neuron model is that an input, **x**, together with a bias, *b* is weighted by, **w**, and then summarized together. The bias, *b*, is a scalar value whereas the input **x** and the weights **w** are vector valued, i.e., **x** ∈ ℝ^*n*^ and **w** ∈ ℝ^*n*^ with *n* ∈ ℕ corresponding to the dimension of the input. Note that the bias term is not always present but is sometimes omitted. The sum of these terms, i.e., *z* = **w**^*T*^**x** + *b* forms then the argument of an activation function, ϕ, resulting in the output of the neuron model,

(1) $$\begin{array}{l} y=\phi (z)=\phi {(\text{w}}^{T}\text{x}+b). \end{array}$$

Considering only the argument of ϕ one obtains a linear discriminant function (Webb and Copsey, 2011).

The activation function, ϕ, (also known as unit function or transfer function) performs a non-linear transformation of *z*. In Table 1, we give an overview of frequently used activation functions.

The ReLU activation function is called Rectified Linear Unit or rectifier (Nair and Hinton, 2010). The ReLU activation function is the most popular activation function for deep neural networks. Another useful activation function is the softmax function (Lawrence et al., 1997):

(2) $$\begin{array}{l} y_{i}=\frac{e^{x_{i}}}{\sum_{j}^{n}e^{x_{j}}}. \end{array}$$

The softmax maps a *n*-dimensional vector **x** into a *n*-dimensional vector **y** having the property $\underset{i}{\sum }y_{i}=1$. Hence, the components of **y** represent probabilities for each of the *n* elements. The softmax is often used in the final layer of a network. If the Heaviside step function is used as activation function, the neuron model is known as *perceptron* (Rosenblatt, 1957).

Usually, the model neuron shown in Figure 2A is represented in a more ergonomic way by limiting the focus on its key elements. In Figure 2B, we show such a representation that highlights merely the input part.

### 3.2. Feedforward Neural Networks

**Figure 3:**
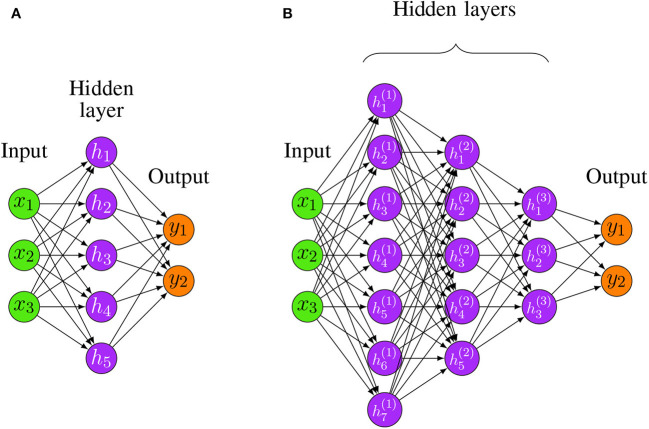
Two examples for Feedforward Neural Networks. **(A)** A shallow FFNN. **(B)** A Deep Feedforward Neural Network (D-FFNN) with 3 hidden layers.

In order to build neural networks (NNs), the neurons need to be connected with each other. The simplest architecture of a NN is a feedforward structure. In Figures 3A,B, we show examples for a shallow and a deep architecture.

In general, the depth of a network denotes the number of non-linear transformations between the separating layers whereas the dimensionality of a hidden layer, i.e., the number of hidden neurons, is called its width. For instance, the shallow architecture in Figure 3A has a depth of 2 whereas Figure 3B has a depth of 4 [total number of layers minus one (input layer)]. The required number to call a Feedforward Neural Network (FFNN) architecture deep is debatable, but architectures with more than two hidden layers are commonly considered as deep (Yoshua, 2009).

A Feedforward Neural Network, also called a Multilayer Perceptron (MLP), can use linear or non-linear activation functions (Goodfellow et al., 2016). Importantly, there are no cycles in the NN that would allow a direct feedback. Equation (3) defines how the output of a MLP is obtained from the input (Webb and Copsey, 2011).

(3) $$\begin{array}{l} f\left(\text{x}\right)=\varphi^{\left(2\right)}\left(W^{\left(2\right)}\varphi^{\left(1\right)}\left(W^{\left(1\right)}\text{x}+{\text{b}}^{\left(1\right)}\right)+{\text{b}}^{\left(2\right)}\right). \end{array}$$

Equation (3) is the discriminant function of the neural network (Webb and Copsey, 2011). For finding the optimal parameters one needs a learning rule. A common approach is to define an error function (or cost function) together with an optimization algorithm to find the optimal parameters by minimizing the error for training data.

### 3.3. Recurrent Neural Networks

The family of Recurrent Neural Network (RNN) models has two subclasses that can be distinguished based on their signal processing behavior. The first contains finite impulse recurrent networks (FRNs) and the second infinite impulse recurrent networks (IIRNs). That difference is that a FRN is given by a directed acyclic graph (DAG) that can be unrolled in time and replaced with a Feedforward Neural Network, whereas an IIRN is a directed cyclic graph (DCG) for which such an unrolling is not possible.

#### 3.3.1. Hopfield Networks

A Hopfield Network (HN) (Hopfield, 1982) is an example for a FRN. A HN is defined as a fully connected network consisting of McCulloch-Pitts neurons. A McCulloch-Pitts neuron is a binary model with an activation function given by

(4) $$s=\text{sgn}(x)=\{\begin{array}{ll} +1 & \text{for }x\geq 0\\ -1 & \text{for }x<0 \end{array}$$

The activity of the neurons *x*_*i*_, i.e.,

(5) $$\begin{array}{l} x_{i}=\text{sgn}\left(\overset{N}{\underset{j=1}{\sum }}w_{ij}x_{j}-\theta_{i}\right) \end{array}$$

is either updated synchronously or asynchronously. To be precise, *x*_*j*_ refers to $x_{j}^{t}$ and *x*_*i*_ to $x_{i}^{t+1}$ (time progression).

Hopfield Networks have been introduced to serve as a model of a content-addressable (“associative”) memory, i.e., for storing patterns. In this case, it has been shown that the weights are obtained by

(6) $$\begin{array}{l} w_{ij}=\overset{P}{\underset{k=1}{\sum }}t_{i}\left(k\right)t_{j}\left(k\right) \end{array}$$

whereas *P* is the number of patterns, *t*(*k*) is the k-th pattern and *t*_*i*_(*k*) its i-th component. From Equation (6), one can see that the weights are symmetrical. An interesting question in this context is what is the maximal value of *P* or *P*/*N*, called the network capacity (here *N* is the total number of patterns). In Hertz et al. (1991) it was shown that the network capacity is ≈0.138. It is interesting to note that the neurons in a Hopfield Network cannot be distinguished as input neurons, hidden neurons and output neurons because at the beginning every neuron is an input neuron, during the processing every neuron is a hidden neuron and at the end every neuron is an output neuron.

#### 3.3.2. Boltzmann Machine

A Boltzmann Machine (Hinton and Sejnowski, 1983) can be described as a *noisy* Hopfield network because it uses a probabilistic activation function

(7) $$\begin{array}{l} p\left(s_{i}=1\right)=\frac{1}{1+\text{exp}\left(-x_{i}\right)} \end{array}$$

whereas *x*_*i*_ is obtained as in Equation (5). This model is important because it is one of the first neural networks that uses hidden units (latent variables). For learning the weights, the Contrastive Divergence algorithm (see Algorithm 9) can be used to train Boltzmann Machines. Put simply, Boltzmann Machines are neural networks consisting of two layers—a visible layer and a hidden layer. Each edge between the two layers is undirected, implying that information can flow in a bi-directional way. The whole network is fully connected, which means that each neuron in the network is connected to all other neurons via undirected edges (see Figures 8A,B).

### 3.4. An Overview of Network Architectures

There is a large variety of different network architectures used as deep learning models. The following Table 2 does not aim to provide a comprehensive list, but it includes the most popular models currently used (Yoshua, 2009; LeCun et al., 2015).

**Table 2:** List of popular deep learning models, available learning algorithms (unsupervised, supervised) and software implementations in R or python.

**Model**	**Unsupervised**	**Supervised**	**Software**
Autoencoder	✓		Keras (Chollet, 2015), R: dimRed (Kraemer et al., 2018), h2o (Candel et al., 2015), RcppDL (Kou and Sugomori, 2014)
Convolutional Deep Belief Network (CDBN)	✓	✓	R & python: TensorFlow (Abadi et al., 2016), Keras (Chollet, 2015), h2o (Candel et al., 2015)
Convolutional Neural Network (CNN)	✓	✓	R & python: Keras (Chollet, 2015) MXNet (Chen et al., 2015), Tensorflow (Abadi et al., 2016), h2O (Candel et al., 2015), fastai (python) (Howard and Gugger, 2018)
Deep Belief Network (DBN)	✓	✓	RcppDL (R) (Kou and Sugomori, 2014), python: Caffee (Jia et al., 2014), Theano (Theano Development Team, 2016), Pytorch (Paszke et al., 2017), R & python: TensorFlow (Abadi et al., 2016), h2O (Candel et al., 2015)
Deep Boltzmann Machine (DBM)		✓	python: boltzmann-machines (Bondarenko, 2017), pydbm (Chimera, 2019)
Denoising Autoencoder (dA)	✓		Tensorflow (R, python) (Abadi et al., 2016), Keras (R, python) (Chollet, 2015), RcppDL (R) (Kou and Sugomori, 2014)
Long short-term memory (LSTM)		✓	rnn (R) (Quast, 2016), OSTSC (R) (Dixon et al., 2017), Keras (R and python) (Chollet, 2015), Lasagne (python) (Dieleman et al., 2015), BigDL (python) (Dai et al., 2018), Caffe (python) (Jia et al., 2014)
Multilayer Perceptron (MLP)		✓	SparkR (R) (Venkataraman et al., 2016), RSNNS (R) (Bergmeir and Benítez, 2012), keras (R and python) (Chollet, 2015), sklearn (python) (Pedregosa et al., 2011), tensorflow (R and python) (Abadi et al., 2016)
Recurrent Neural Network (RNN)		✓	RSNNS (R) (Bergmeir and Benítez, 2012), rnn (R) (Quast, 2016), keras (R and python) (Chollet, 2015)
Restricted Boltzmann Machine (RBM)	✓	✓	RcppDL (R) (Kou and Sugomori, 2014), deepnet (R) (Rong, 2014), pydbm (python) (Chimera, 2019), sklearn (python) (Chimera, 2019), Pylearn2 (Goodfellow et al., 2013), TheanoLM (Enarvi and Kurimo, 2016)

It is interesting to note that some of the models in Table 2 are composed by other networks. For instance, CDBNs are based on RBMs and CNNs (Lee et al., 2009); DBMs are based on RBMs (Salakhutdinov and Hinton, 2009); DBNs are based on RBMs and MLPs; dAEs are stochastic Autoencoders that can be stacked on top of each other to build stacked denoising Autoencoders (SdAEs).

In the following sections, we discuss the major core architectures Deep Feedforward Neural Networks (D-FFNN), Convolutional Neural Networks (CNNs), Deep Belief Networks (DBNs), Autoencoders (AEs), and Long Short-Term Memory networks (LSTMs) in more detail.

## 4. Deep Feedforward Neural Networks

It can be proven that a Feedforward Neural Network with one hidden layer and a finite number of neurons can approximate any continuous function on a compact subset of ℝ^*n*^ (Hornik, 1991). This is called the *universal approximation theorem*. The reason for using a FFNN with more than one hidden layer is that the universal approximation theorem does not provide information on how to learn such a network, which turned out to be very difficult. A related issue that contributes to the difficulty of learning such networks is that their width can become exponentially large. Interestingly, the universal approximation theorem can also be proven for FFNN with many hidden layers and a bounded number of hidden neurons (Lu et al., 2017) for which learning algorithms have been found. Hence, D-FFNNs are used instead of (shallow) FFNNs for practical reasons of learnability.

Formally, the idea of approximating an unknown function f^*^ can be written as

(8) $$y=f^{*}(x)\approx f(x,w)\approx \phi (x^{T}w).$$

Here ***f*** is a function from a specific family that depends on the parameters θ, and ***ϕ*** is a non-linear activation function with one layer. For many hidden layers ***ϕ*** has the form

(9) $$\begin{array}{l} \phi =\phi^{\left(n\right)}\left(\ldots \phi^{\left(2\right)}\left(\phi^{\left(1\right)}\left(\text{x}\right)\right)\ldots \right). \end{array}$$

Instead of *guessing* the correct family of functions from which ***f*** should be chosen, D-FFNNs learn this function by approximating it via ***ϕ***, which itself is approximated by the *n* hidden layers.

The practical learning of the parameters of a D-FFNN (see Figure 3B) can be accomplished with the backpropagation algorithm, although for computational efficiency nowadays the Stochastic Gradient Descent is used (Bottou, 2010). The Stochastic Gradient Descent calculates a gradient for a set of randomly chosen training samples (batch) and updates the parameters for this batch sequentially. This results in a faster learning. A drawback is an increase in imprecision. However, for data sets with a large number of samples (big data), the speed advantage outweighs this drawback.

## 5. Convolutional Neural Networks

A Convolutional Neural Network (CNN) is a special Feedforward Neural Network utilizing convolution, ReLU and pooling layers. Standard CNNs are normally composed of several Feedforward Neural Network layers including convolution, pooling, and fully-connected layers.

Typically, in traditional ANNs, each neuron in a layer is connected to all neurons in the next layer, whereas each connection is a parameter in the network. This can result in a very large number of parameters. Instead of using fully connected layers, a CNN uses a local connectivity between neurons, i.e., a neuron is only connected to nearby neurons in the next layer. This can significantly reduce the total number of parameters in the network.

**Figure 4:**
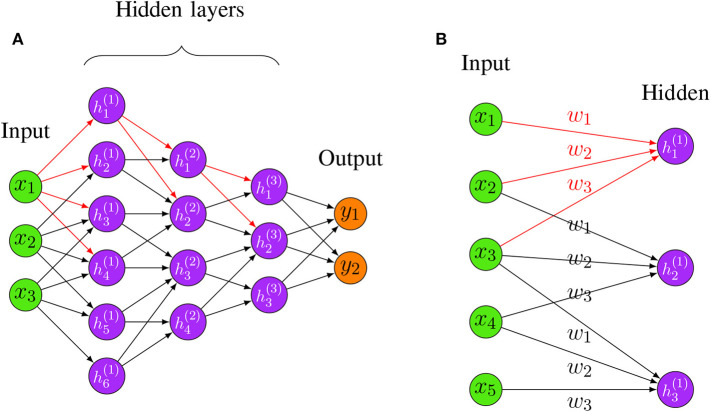
**(A)** An example for a Convolutional Neural Network. The red edges highlight the fact that hidden layers are connected in a “local” way, i.e., only very few neurons connect the succeeding layers. **(B)** An example for shared weights and local connectivity in CNN. The red edges highlight the fact that hidden layers are connected in a “local” way, i.e., only very few neurons connect the succeeding layers. The labels *w*_1_,*w*_2_,*w*_3_ indicate the assigned weight for each connection, three hidden nodes share the same set of weights *w*_1_,*w*_2_,*w*_3_ when connecting to three local patches.

Furthermore, all the connections between local receptive fields and neurons use a set of weights, and we denote this set of weights as a kernel. A kernel will be shared with all the other neurons that connect to their local receptive fields, and the results of these calculations between the local receptive fields and neurons using the same kernel will be stored in a matrix denoted as *activation map*. The sharing property is referred to as weight sharing of CNNs (Le Cun, 1989). Consequently, different kernels will result in different activation maps, and the number of kernels can be adjusted with hyper-parameters. Thus, regardless of the total number of connections between the neurons in a network, the total number of weights corresponds only to the size of the local receptive field, i.e., the size of the kernel. This is visualized in Figure 4B, where the total number of connections between the two layers is 9 but the size of the kernel is only 3.

By combining weight sharing and the local connectivity property, a CNN is able to handle data with high dimensions. See Figure 4A for a visualization of a CNN with three hidden layers. In Figure 4A, the red edges highlight the locality property of hidden neurons, i.e., only very few neurons connect to the succeeding layers. This locality property of CNN makes the network sparse compared to a FFNN which is fully connected.

### 5.1. Basic Components of CNN

#### 5.1.1. Convolutional Layer

A convolutional layer is an essential part in building a convolutional neural network. Similar to a hidden layer of an ordinary neural network, a convolutional layer has the same goal, which is to convert the input into a representation of a more abstract level. However, instead of using a full connectivity, the convolutional layer uses a local connectivity to perform the calculations between input and the hidden neurons. A convolutional layer uses at least one kernel to slide across the input, performing a convolution operation between each input region and the kernel. The results are stored in the activation maps, which can be seen as the output of the convolutional layer. Importantly, the activation maps can contain features extracted by different kernels. Each kernel can act as a feature extractor and will share its weights with all neurons.

For the convolution process, some spatial arguments need to be defined in order to produce the activation maps of a certain size. Essential attributes include:

1. Size of kernels (N). Each kernel has a window size, which is also referred to as receptive field. The kernel will perform a convolution operation with a region matching its window size from the input, and produce results in its activation map.
2. Stride (S). This parameter defines the number of pixels the kernel will move for the next position. If it is set to 1, each kernel will make convolution operations around the input volume and then shift 1 pixel at a time until it reaches the specified border of the input. Hence, the stride can be used to downsize the dimension of the activation maps as the larger the stride the smaller the activation maps.
3. Zero-padding (P). This parameter is used to specify how many zeros one wants to pad around the border of the input. This is very useful for preserving the dimension of the input.

These three parameters are the most common hyper-parameters used for controlling the output volume of a convolutional layer. Specifically, for an input of dimension *W*_*input*_ × *H*_*input*_ × *Z*, for the hyper-parameters size of the kernel (N), Stride (S), and Zero-padding (P) the dimension of the activation map, i.e., *W*_*out*_ × *H*_*out*_ × *D* can be calculated by:

(10) $$\begin{array}{l} W_{out}=\frac{\left(W_{input}-N+2P\right)}{S+1}\\ H_{out}=\frac{\left(H_{input}-N+2P\right)}{S+1}\\ \;D=Z \end{array}$$

**Figure 5:**
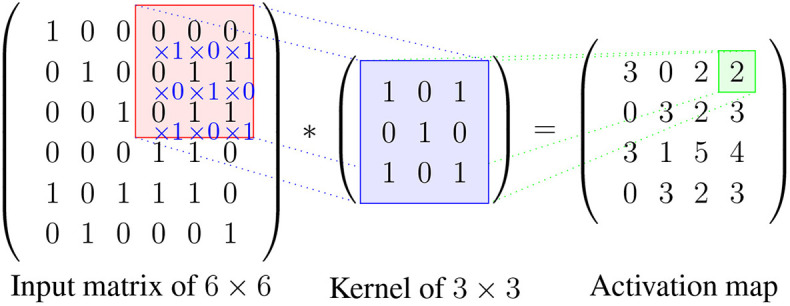
An example for calculating the values in the activation map. Here, the stride is 1 and the zero-padding is 0. The kernel slides by 1 pixel at a time from left to right starting from the left top position, after reaching the boarder the kernel will start from the second row and repeat the process until the whole input is covered. The red area indicates the local patch to be convoluted with the kernel, and the result is stored in the green field in the activation map.

An example of how to calculate the result between an input matrix and a kernel can be seen in Figure 5.

The shared weights and the local connectivity help significantly in reducing the total number of parameters of the network. For example, assuming that an input has dimension 100 × 100 × 3, and that the convolutional layer and the number of kernels is 2 and each kernel has a local receptive field of size 4, then the dimension of each kernel is 4 × 4 × 3 (3 is the depth of the kernel which will be the same as the depth of the input volume). For 100 neurons in the layer there will be in total only 4 × 4 × 3 × 2 = 96 parameters in this layer because all the 100 neurons will share the same weights for each kernel. This considers only the number of kernels and the size of the local connectivity but does not depend on the number neurons in the layer.

In addition to reducing the number of parameters, shared weights and a local connectivity are important in processing images efficiently. The reason therefore is that local convolutional operations in an image result in values that contain certain characteristics of the image, because in images local values are generally highly correlated and the statistics formed by the local values are often invariant in the location (LeCun et al., 2015). Hence, using a kernel that shares the same weights can detect patterns from all the local regions in the image, and different kernels can extract different types of patterns from the image.

A non-linear activation function (for instance ReLu, tanh, sigmoid, etc.) is often applied to the values from the convolutional operations between the kernel and the input. These values are stored in the activation maps, which will be later passed to the next layer of the network.

#### 5.1.2. Pooling Layer

A pooling layer is usually inserted between a convolutional layer and the following layer. Pooling layers aim at reducing the dimension of the input with some pre-specified pooling method, resulting in a smaller input by conserving as much information as possible. Also, a pooling layer is able to introduce spatial invariance into the network (Scherer et al., 2010), which can help to improve the generalization of the model. In order to perform pooling, a pooling layer uses stride, zero-padding, and a pooling window size as hyper-parameters. The pooling layer will scan the entire input with the specified pooling window size in the same manner as the kernel in a convolutional layer. For instance, using a stride of 2, window size of 2 and 0 zeros-padding for pooling will half the size of the input dimension.

There are many types of pooling methods, e.g., averaging-pooling, min-pooling and some advanced pooling methods, such as fractional max-pooling and stochastic pooling. The most common used pooling method is max-pooling, as it has been shown to be superior in dealing with images by capturing invariances efficiently (Scherer et al., 2010). Max-pooling extracts the maximum value within each specified sub-window across the activation map. The max-pooling can be formulated as *A*_*i, j, k*_ = *max*(*R*_*i*−*n*:*i*+*n, j*−*n*:*j*+*n, k*_), where *A*_*i, j, k*_ is the maximum activation value from the matrix *R* of size *n* × *n* centered at index *i, j* in the *kth* activation map with *n* is the window size.

#### 5.1.3. Fully-Connected Layer

A fully-connected layer is the basic hidden layer unit in FFNN (see section 3.2). Interestingly, also for traditional CNN architectures, a fully connected layer is often added between the penultimate layer and the output layer to further model non-linear relationships of the input features (Krizhevsky et al., 2012b; Simonyan and Zisserman, 2014; Szegedy et al., 2015). However, recently the benefit of this has been questioned because of the many parameters introduced by this, leading potentially to overfitting (Simonyan and Zisserman, 2014). As a result, more and more researchers started to construct CNN architecture without such a fully connected layer using other techniques like max-over-time pooling (Lin et al., 2013; Kim, 2014) to replace the role of linear layers.

### 5.2. Important Variants of CNN

#### 5.2.1. VGGNet

VGGNet (Simonyan and Zisserman, 2014) was a pioneer in exploring how the depth of the network influences the performance of a CNN. VGGNet was proposed by the Visual Geometry Group and Google DeepMind, and they studied architectures with a depth of 19 (e.g., compared to 11 for AlexNet Krizhevsky et al., 2012b).

VGG19 extended the network from eight weight layers (a structure proposed by AlexNet) to 19 weights layers by adding 11 more convolutional layers. In total, the parameters increased from 61 million to 144 million, however, the fully connected layer takes up most of the parameters. According to their reported results, the error rate dropped from 29.6 to 25.5 regrading top-1 val.error (percentage of times the classifier did not give the correct class with the highest score) on the ILSVRC dataset, and from 10.4 to 8.0 regarding top-5 val.error (percentage of times the classifier did not include the correct class among its top 5) on the ILSVRC dataset in ILSVRC2014. This indicates that a deeper CNN structure is able to achieve better results than shallower networks. In addition, they stacked multiple 3 × 3 convolutional layers without a pooling layer placed in between to replace the convolutional layer with a large filter sizes, e.g., 7 × 7 or 11 × 11. They suggested such an architecture is capable of receiving the same receptive fields as those composed of larger filter sizes. Consequently, two stacked 3 × 3 layers can learn features from a 5 × 5 receptive field, but with less parameters and more non-linearity.

#### 5.2.2. GoogLeNet With Inception

The most intuitive way for improving the performance of a Convolutional Neural Network is to stack more layers and add more parameters to the layers (Simonyan and Zisserman, 2014). However, this will impose two major problems. One is that too many parameters will lead to overfitting, and the other is that the model becomes hard to train.

GoogLeNet (Szegedy et al., 2015) was introduced by Google. Until the introduction of inception, traditional state-of-the-art CNN architectures mainly focused on increasing the size and depth of the neural network, which also increased the computation cost of the network. In contrast, GoogLeNet introduced an architecture to achieve state-of-the-art performance with a light-weight network structure.

**Figure 6:**
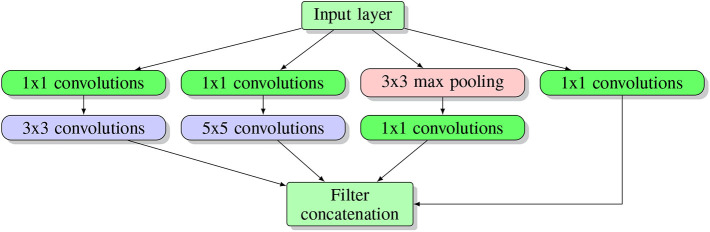
Inception block structure. Here multiple blocks are stacked on top of each other, forming the input layer for the next block.

The idea underlying an inception network architecture is to keep the network as sparse as possible while utilizing the fast matrix computation feature provided by a computer. This idea facilitates the first inception structure (see Figure 6).

As one can see in the Figure 6, several parallel layers including 1 × 1 convolution and 3 × 3 max pooling operate at the same level on the input. Each tunnel (namely one separated sequential operation) has a different child layer, including 3 × 3 convolutions, 5 × 5 convolutions and 1 × 1 convolution layer. All the results from each tunnel are concatenated together at the output layer. In this architecture, a 1x1 convolution is used to downscale the input image while reserving input information (Lin et al., 2013). They argued that concatenating all the features extracted by different filters corresponds to the idea that image information should be processed at different scales and only the aggregated features should be sent to the next level. Hence, the next level can extract features from different scales. Moreover, this sparse structure introduced by an inception block requires much fewer parameters and, hence, is much more efficient.

By stacking the inception structure throughout the network, GoogLeNet won first place in the classification task of ILSVRC2014, demonstrating the quality of the inception structure. Followed by the inception v1, inception v2, v3, and the latest version v4 were introduced. Each generation introduced some new features, making the network faster, more light-weight and more powerful.

#### 5.2.3. ResNet

In principle, CNNs with a deeper structure perform better than shallow ones (Simonyan and Zisserman, 2014). In theory, deeper networks have a better ability to represent high level features from the input, therefore improving the accuracy of predictions (Donahue et al., 2014). However, one cannot simply stack more and more layers. In the paper (He et al., 2016), the authors observed the phenomena that more layers can actually hurt the performance. Specifically, in their experiment, network A had *N* layers, and network B had *N* + *M* layers, while the initial *N* layers had the same structure. Interestingly, when training on the CIFAR-10 and ImageNet dataset, network B showed a higher training error than network B. In theory, the extra *M* layers should result in a better performance, but instead they obtained higher errors which cannot be explained by overfitting. The reason for this is that the loss is getting optimized to local minima, which is different to the vanishing gradient phenomena. This is referred to as the degradation problem (He et al., 2016).

**Figure 7:**
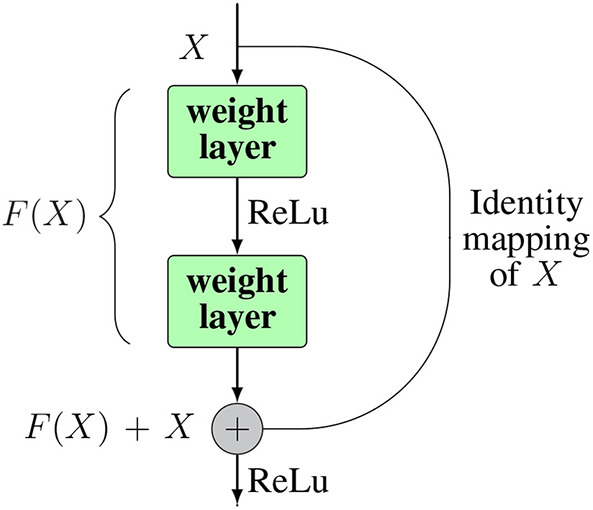
The structure of a residual block. Inside a block there can be as many weight layers as desired.

ResNet (He et al., 2016) was introduced to overcome the degradation problem of CNNs to push the depth of a CNN to its limit. In (He et al., 2016), the authors proposed a novel structure of a CNN, which is in theory capable of being extended to an infinite depth without losing accuracy. In their paper, they proposed a deep residual learning framework, which consists of multiple residual blocks to address the degradation problem. The structure of a residual block is shown in the Figure 7.

Instead of trying to learn the desired underlying mapping *H*(*x*) from each few stacked layers, they used an identity mapping for input *x* from input to the output of the layer, and then let the network learn the residual mapping *F*(*x*) = *H*(*x*) − *x*. After adding the identity mapping, the original mapping can be reformulated as *H*(*x*) = *F*(*x*) + *x*. The identity mapping is realized by making shortcut connections from the input node directly to the output node. This can help to address the degradation problem as well as the vanishing (exploding) gradient issue of deep networks. In extreme cases, deeper layers can just learn the identity map of the input to the output layer, by simply calculating the residuals as 0. This enables the ability for a deep network to perform at least not worse than shallow ones. Also, in practice, the residuals are never 0, which makes it possible for very deeper layers to always learn something new from the residuals therefore producing better results. The implementation of ResNet helped to push the layers of CNNs to 152 by stacking so-called residual blocks through out the network. ResNet achieved the best result in the ILSVRC2016 competition, with an error rate of 3.57.

## 6. Deep Belief Networks

A Deep Belief Network (DBN) is a model that combines different types of neural networks with each other to form a new neural network model. Specifically, DBNs integrate Restricted Boltzmann Machines (RBMs) with Deep Feedforward Neural Networks (D-FFNN). The RBMs form the input unit whereas the D-FFNNs form the output unit. Frequently, RBMs are stacked on top of each other, which means more than one RBM is used sequentially. This adds to the depth of the DBN.

Due to the different nature of the networks RBM and D-FFNN, two different types of learning algorithms are used. Practically, the Restricted Boltzmann Machines are used for initializing a model in an unsupervised way. Thereafter, a supervised method is applied for the fine tuning of the parameters (Yoshua, 2009). In the following, we describe these two phases of the training of a DBN in more detail.

### 6.1. Pre-training Phase: Unsupervised

Theoretically, neural networks can be learned by using supervised methods only. However, in practice it was found that such a learning process can be very slow. For this reason, unsupervised learning is used to initialize the model parameters. The standard neural network learning algorithm (backpropagation) was initially only able to learn shallow architectures. However, by using a Restricted Boltzmann Machine for the unsupervised initialization of the parameters one obtains a more efficient training of the neural network (Hinton et al., 2006).

**Figure 8:**
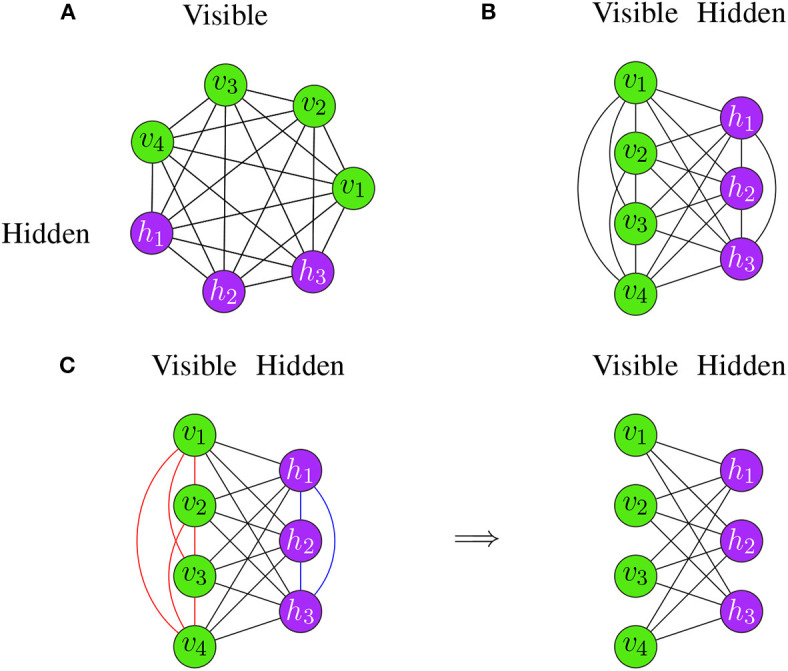
Examples for Boltzmann Machines. **(A)** The neurons are arranged on a circle. **(B)** The neurons are separated according to their type. Both Boltzmann Machines are identical and differ only in their visualization. **(C)** Transition from a Boltzmann Machine (left) to a Restricted Boltzmann Machine (right).

A Restricted Boltzmann Machine is a special type of a Boltzmann Machine (BM), see section 3.3.2. The difference between a Restricted Boltzmann Machine and a Boltzmann Machine is that Restricted Boltzmann Machines (RBMs) have constraints in the connectivity of their structure (Fischer and Igel, 2012). Specifically, there can be no connections between nodes in the same layer. For an example, see Figure 8C.

The values of neurons, **v**, in the visible layer are known, but the neuron values, **h**, in the hidden layer are unknown. The parameters of the network are learned by defining an energy function, *E*, of the model which is then minimized.

Frequently, a RBM is used with binary values, i.e., *v*_*i*_ ∈ {0, 1} and *h*_*i*_ ∈ {0, 1}. The energy function for such a network is given by (Hinton, 2012):

(11) $$\begin{array}{l} E\left(\text{v},\text{h}\right)=-\overset{m}{\underset{i}{\sum }}a_{i}v_{i}-\overset{n}{\underset{j}{\sum }}b_{j}h_{j}-\overset{m}{\underset{i}{\sum }}\overset{n}{\underset{j}{\sum }}v_{i}h_{j}w_{i,j} \end{array}$$

whereas Θ = {**a, b**, *W*} is the set of model parameters.

Each configuration of the system corresponds to a probability defined via the Boltzmann distribution in Equation (11):

(12) $$\begin{array}{l} p\left(\text{v},\text{h}\right)=\frac{1}{Z}e^{-E\left(\text{v},\text{h}\right)} \end{array}$$

In Equation (12), *Z* is the partition function given by:

(13) $$\begin{array}{l} Z=\underset{\text{v},\text{h}}{\sum }e^{-E\left(\text{v},\text{h}\right)} \end{array}$$

The probability for the network assigning to a visible vector **v** is given by summing over all possible hidden vectors:

(14) $$\begin{array}{l} p\left(\text{v}\right)=\frac{1}{Z}\underset{\text{h}}{\sum }e^{-E\left(\text{v},\text{h}\right)} \end{array}$$

Maximum-likelihood estimation (MLE) is used for estimating the optimal parameters of the probabilistic model (Hayter, 2012). For a training data set $D=D_{train}=\left\{{\text{v}}_{1},\ldots ,{\text{v}}_{l}\right\}$ consisting of *l* patterns, assuming that the patterns are iid (independent and identical) distributed, the log-likelihood function is given by:

(15) $$\begin{array}{l} L\left(\theta \right)=\text{ln }L\left(\theta |D\right)=\text{ln }\overset{l}{\underset{i=1}{\prod }}p\left({\text{v}}_{i}|\theta \right)=\overset{l}{\underset{i=1}{\sum }}\text{ln }p\left({\text{v}}_{i}|\theta \right) \end{array}$$

For simple cases, one may be able to find an analytical solution for Equation (15) by solving $\frac{\partial }{\partial \theta }\text{ln }L\left(\theta |D\right)=0$. However, usually the parameters need to be found numerically. For this, the gradient of the log-likelihood is a typical approach for estimating the optimal parameters:

$$\begin{array}{l} \theta^{\left(t+1\right)}=\theta^{\left(t\right)}+\Delta \theta^{\left(t\right)}=\theta^{\left(t\right)}+\eta \frac{\partial L\left(\theta^{t}\right)}{\partial \theta^{\left(t\right)}}-\lambda \theta^{\left(t\right)}+\nu \Delta \theta^{\left(t-1\right)} \end{array}$$

In Equation (16), the constant, η, in front of the gradient is the learning rate and the first regularization term, −λθ^(*t*)^, is the weight-decay. The weight-decay is used to constrain the optimization problem by penalizing large values of θ (Hinton, 2012). The parameter λ is also called *the weight-cost*. The second regularization term in Equation (16) is called momentum. The purpose of the momentum is to make learning faster and to reduce possible oscillations. Overall, this should stabilize the learning process.

For the optimization, the Stochastic Gradient Ascent (SGA) is utilized using *mini-batches*. That means one selects randomly a number of samples from the training set, *k*, which are much smaller than the total sample size, and then estimates the gradient. The parameters, θ, are then updated for the mini-batch. This process is repeated iteratively until an epoch is completed. An epoch is characterized by using the whole training set once. A common problem is encountered when using mini-batches that are too large, because this can slow down the learning process considerably. Frequently, *k* is chosen between 10 and 100 (Hinton, 2012).

Before the gradient can be used, one needs to approximate the gradient of Equation (16). Specifically, the derivatives with respect to the parameters can be written in the following form:

(17) $$\{\begin{array}{l} \frac{\partial ℒ(\theta |\text{v})}{\partial w_{ij}}=p(H_{j}=1|\text{v})v_{i}-\sum_{\text{v}}p(\text{v})p(H_{j}=1|\text{v})v_{i}\\ \frac{\partial ℒ(\theta |\text{v})}{\partial a_{i}}=v_{i}-\sum_{\text{v}}p(\text{v})v_{i}\\ \frac{\partial ℒ(\theta |\text{v})}{\partial b_{j}}=p(H_{j}=1|\text{v})-\sum_{\text{v}}p(\text{v})p(H_{j}=1|\text{v}) \end{array}$$

In Equation (17), *H*_*i*_ denotes the value of hidden unit *i* and *p*(**v**) is the probability defined in Equation (14). For the conditional probability, one finds

(18) $$\begin{array}{l} p\left(H_{j}=1|\text{v}\right)=\sigma \left(\overset{n}{\underset{j=1}{\sum }}w_{ij}v_{i}+b_{j}\right) \end{array}$$

and correspondingly

(19) $$\begin{array}{l} p\left(V_{i}=1|\text{h}\right)=\sigma \left(\overset{m}{\underset{i=1}{\sum }}w_{ij}h_{j}+a_{i}\right) \end{array}$$

**Figure 9:**
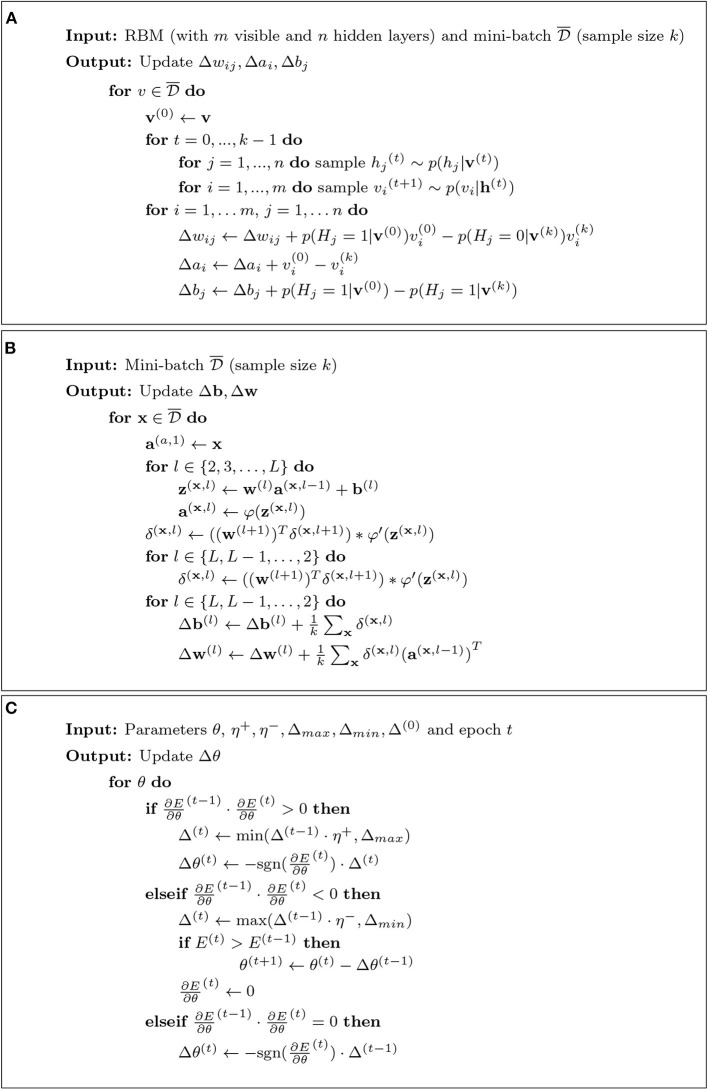
**(A)** Contrastive Divergence k-step algorithm using Gibbs sampling. **(B)** Backpropagation algorithm. **(C)** iRprop^+^ algorithm.

Using the above equations in the presented form would be inefficient because these equations require a summation over all visible vectors. For this reason, the Contrastive Divergence (CD) method is used for increasing the speed for the estimation of the gradient. In Figure 9A, we show pseudocode of the CD algorithm.

The CD uses Gibbs sampling for drawing samples from conditional distributions, so that the next value depends only on the previous one. This generates a Markov chain (Hastie et al., 2009). Asymptotically, for *k* → ∞ the distribution becomes the true stationary distribution. In this case, the *CD* → *ML*. Interestingly, already *k* = 1 can lead to satisfactory approximations for the pre-training (Carreira-Perpinan and Hinton, 2005).

**Figure 10:**
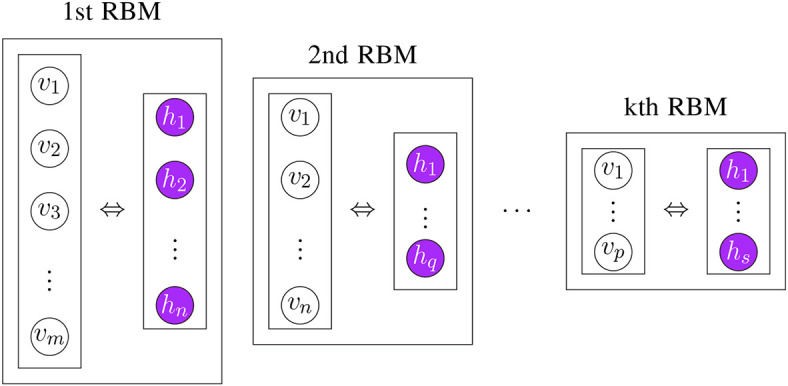
Visualizing the stacking of RBMs in order to learn the parameters Θ of a model in an unsupervised way.

In general, pre-training of DBNs consists of stacking RBMs. That means the next RBM is trained using the hidden layer of the previous RBM as visible layer. This initializes the parameters for each layer (Hinton and Salakhutdinov, 2006). Interestingly, the order of this training is not fixed but can vary. For instance, first, the last layer can be trained and then the remaining layers can be trained (Hinton et al., 2006). In Figure 10, we show an example for the stacking of RBMs.

### 6.2. Fine-Tuning Phase: Supervised

After the initialization of the parameters of the neural network, as described in the previous step, these can now be fine-tuned. For this step, a supervised learning approach is used, i.e., the labels of the samples, omitted in the pre-training phase, are now utilized.

For learning the model, one minimizes an error function (also called loss function or sometimes objective function). An example for such an error function is the mean squared error (MSE).

(20) $$\begin{array}{l} E=\frac{1}{2n}\overset{n}{\underset{i=1}{\sum }}{∥{\text{o}}_{i}-{\text{t}}_{i}∥}^{2} \end{array}$$

In Equation (20), **o**_*i*_ = ***ϕ***(**x**_*i*_) is the i^th^ output from the network function ***ϕ***:ℝ^*m*^ → ℝ^*n*^ given the i^th^ input **x**_*i*_ from the training set $D=D_{train}=\left\{\left({\text{x}}_{1},{\text{t}}_{1}\right),\ldots \left({\text{x}}_{l},{\text{t}}_{l}\right)\right\}$ and **t**_*i*_ is the target output.

Similarly, for maximizing the log-likelihood function of a RBM (see Equation 16), one uses gradient descent to find the parameters that minimize the error function.

(21) $$\begin{array}{l} \theta^{\left(t+1\right)}=\theta^{\left(t\right)}-\Delta \theta^{\left(t\right)}=\theta^{\left(t\right)}-\eta \frac{\partial E}{\partial \theta^{\left(t\right)}}-\lambda \theta^{\left(t\right)}+\nu \Delta \theta^{\left(t-1\right)} \end{array}$$

Here, the parameters (η, λ and ν) have the same meaning as explained above. Again, the gradient is typically not used for the entire training data D, but instead smaller batches are used via the *Stochastic Gradient Descent* (SGD).

The gradient of the RBM log-likelihood can be approximated using the CD algorithm (see Figure 9A). For this, the *backpropagation algorithm* is used (LeCun et al., 2015).

Let us denote by ${a_{i}}^{l}$ the activation of the ith unit in the lth layer (*l* ∈ {2, …, *L*}), $b_{i}^{t}$ the corresponding bias and $w_{ij}^{l}$ the weight for the edge between the jth unit of the (*l* − 1)th layer and the ith unit of the lth layer. For activation function, φ, the activation of the lth layer with the (l - 1)th layer as input is **a**^*l*^ = φ(**z**^(*l*)^) = φ(**w**^(*l*)^**a**^(*l*−1)^ + **b**^(*l*)^).

Application of the chain rule leads to (Nielsen, 2015):

(22) $$\{\begin{array}{l} \delta^{\left(L\right)}=\nabla_{\text{a}}E\cdot \varphi^{\prime }({\text{z}}^{\left(L\right)})\\ \delta^{\left(l\right)}=({\left({\text{w}}^{\left(l+1\right)}\right)}^{T}\delta^{\left(l+1\right)})\cdot \varphi^{\prime }({\text{z}}^{\left(l\right)})\\ \frac{\partial E}{\partial b_{i}^{\left(l\right)}}=\delta_{i}^{\left(l\right)}\\ \frac{\partial E}{\partial w_{ij}^{\left(l\right)}}=x_{j}^{\left(l-1\right)}\delta_{i}^{\left(l\right)} \end{array}$$

In Equation (22), the vector *δ*^*L*^ contains the errors of the output layer (*L*), whereas the vector *δ*^*l*^ contains the errors of the lth layer. Here, · indicates the element-wise product of vectors. From this the gradient of the error of the output layer is given by

(23) $$\nabla_{\text{a}}E=\{\frac{\partial E}{\partial a_{1}^{\left(L\right)}},\ldots ,\frac{\partial E}{\partial a_{k}^{\left(L\right)}}\}.$$

In general, the result of this depends on *E*. For instance, for the MSE we obtain $\frac{\partial E}{\partial a_{j}^{\left(L\right)}}=\left(a_{j}-t_{j}\right)$. As a result, the pseudocode for the backpropagation algorithm can be formulated as shown in Figure 9B (Nielsen, 2015). The estimated gradients from Figure 9B are then used to update the parameters (weights and biases) via SGD (see Equation 21). More updates are performed using mini-batches until all training data have been used (Smolander, 2016).

*The resilient backpropagation algorithm* (Rprop) is a modification of the backpropagation algorithm that was originally introduced to speed up the basic backpropagation (Bprop) algorithm (Riedmiller and Braun, 1993). There exist at least four different versions of Rprop (Igel and Hüsken, 2000) and in Algorithm 9 pseudocode for the iRprop^+^ algorithm (which improves Rprop with weight-backtracking) is shown (Smolander, 2016).

As one can see in Algorithm 9, iRprop^+^ uses information about the sign of the partial derivative from time step (*t* − 1) to make a decision for the update of the parameter. Importantly, the results of comparisons have shown that the iRprop^+^ algorithm is faster than Bprop (Igel and Hüsken, 2000).

It has been shown that the backpropagation algorithm with SGD can learn good neural network models even without a pre-training stage when the training data are sufficiently large (LeCun et al., 2015).

**Figure 11:**
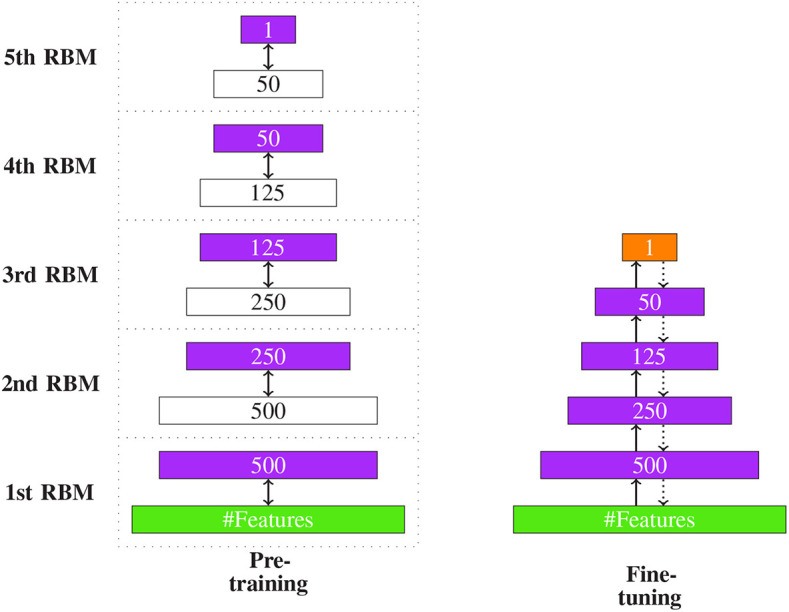
The two stages of DBN learning. **(Left)** The hidden layer (purple) of one RBM is the input of the next RBM. For this reason their dimensions are equal. **(Right)** The two edges in fine-tuning denote the two stages of the backpropagation algorithm: the input feedforwarding and the error backpropagation. The orange layer indicated the output.

In Figure 11, we show an example of the overall DBN learning procedure. The left-hand side shows the pre-training phase and the right-hand side the fine-tuning.

DBNs have been used successfully for many application tasks, e.g., natural language processing (Sarikaya et al., 2014), acoustic modeling (Mohamed et al., 2011), image recognition (Hinton et al., 2006) and computational biology (Zhang S. et al., 2015).

## 7. Autoencoder

An Autoencoder is an unsupervised neural network model used for representation learning, e.g., feature selection or dimension reduction. A common property of autoencoders is that the size of the input and output layer is the same with a symmetric architecture (Hinton and Salakhutdinov, 2006). The underlying idea is to learn a mapping from an input pattern **x** to a new encoding **c** = *h*(**x**), which ideally gives as output pattern the same as the input pattern, i.e., ***x*** ≈ ***y*** = *g*(***c***). Hence, the encoding ***c***, which has usually lower dimension than ***x***, allows to reproduce (or code for) ***x***.

**Figure 12:**
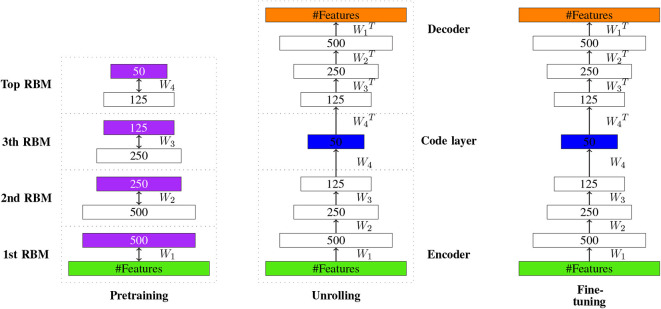
Visualizing the idea of autoencoder learning. The learned new encoding of the input is represented in the code layer (shown in blue).

The construction of Autoencoders is similar to DBNs. Interestingly, the original implementation of an autoencoder (Hinton and Salakhutdinov, 2006) pre-trained only the first half of the network with RBMs and then unrolled the network, creating in this way the second part of the network. Similar to DBNs, a pre-training phase is followed by a fine-tuning phase. In Figure 12, an illustration of the learning process is shown. Here, the coding layer corresponds to the new encoding ***c*** providing, e.g., a reduced dimension of ***x***.

An Autoencoder does not utilize labels and, hence, it is an unsupervised learning model. In applications, the model has been successfully used for dimensionality reduction. Autoencoders can achieve a much better two-dimensional representation of array data, when an adequate amount of data is available (Hinton and Salakhutdinov, 2006). Importantly, PCAs implement a linear transformation, whereas Autoencoders are non-linear. Usually, this results in a better performance. We would like to highlight that there are many extensions of these models, e.g., sparse autoencoder, denoising autoencoder or variational autoencoder (Vincent et al., 2010; Deng et al., 2013; Pu et al., 2016).

## 8. Long Short-Term Memory Networks

Long short-term memory (LSTM) networks were introduced by Hochreiter and Schmidhuber in 1997 (Hochreiter and Schmidhuber, 1997). LSTM is a variant of a RNN that has the ability to address the shortcomings of RNNs which do not perform well, e.g., when handling long-term dependencies (Graves, 2013). Furthermore, LSTMs avoid the gradient vanishing or exploding problem (Hochreiter, 1998; Gers et al., 1999). In 1999, a LSTM with a forget gate was introduced which could reset the cell memory. This improved the initial LSTM and became the standard structure of LSTM networks (Gers et al., 1999). In contrast to Deep Feedforward Neural Networks, LSTMs contain feedback connections. Furthermore, they can not only process single data points, such as vectors or arrays, but sequences of data. For this reason, LSTMs are particularly useful for analyzing speech or video data.

### 8.1. LSTM Network Structure With Forget Gate

**Figure 13:**
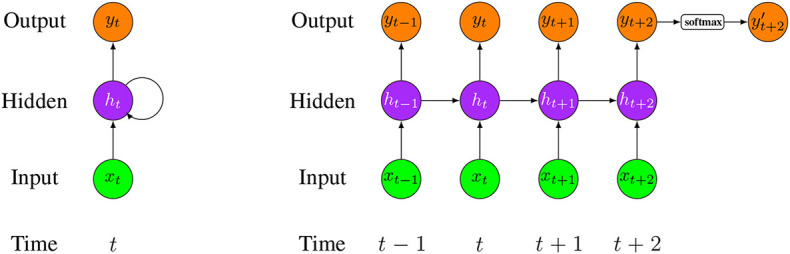
**(Left)** A folded structure of a LSTM network model. **(Right)** An unfolded structure of a LSTM network model. *x*_*i*_ is the input data at time *i* and *y*_*i*_ is the corresponding output (*i* is the time step starting from (*t* − 1)). In this network, only $y_{t+2}^{\prime }$ activated by softmax function is the final network output.

Figure 13 shows an unrolled structure of a LSTM network model (Wang et al., 2016). In this model, the input and output are organized vertically, while information is delivered horizontally over the time series.

In a standard LSTM network, the basic entity is called LSTM unit or a memory block (Gers et al., 1999). Each unit is composed of a cell, the memory part of the unit, and three gates: an input gate, an output gate and a forget gate (also called keep gate) (Gers et al., 2002). A LSTM unit can remember values over arbitrary time intervals and the three gates control the flow of information through the cell. The central feature of a LSTM cell is a part called “constant error carousel” (CEC) (Lipton et al., 2015). In general, a LSTM network is formed exactly like a RNN, except that the neurons in the hidden layers are replaced by memory blocks.

**Figure 14:**
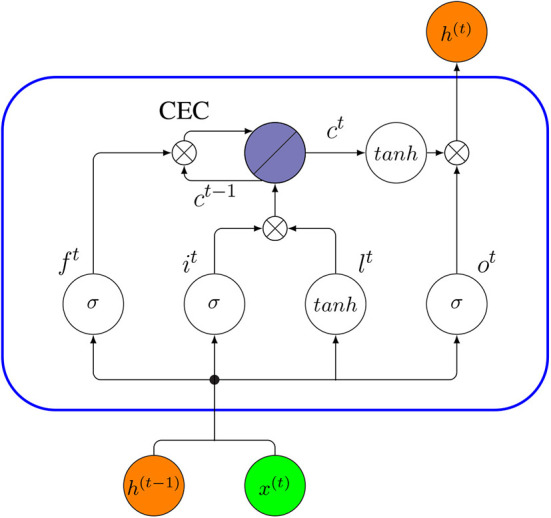
Internal connectivity pattern of a standard LSTM unit (blue rectangle). The output from the previous time step, *h*^(*t*−1)^, and *x*^(*t*)^, are the input to the block at time *t*, then the output *h*^(*t*)^ at time *t* will be an input to the same block in the next time step (*t* + 1).

In the following, we discuss some core concepts and the corresponding technicalities (*W* and *U* stand for the weights and *b* for the bias). In Figure 14, we show a schematic description of a LSTM block with one cell.

- Input gate: A unit with sigmoidal function that controls the flow of information into the cell. It receives its activation from both output of the previous time *h*^(*t*−1)^ and current input *x*^(*t*)^. Under the effect of the sigmoid function, an input gate *i*^*t*^ generates values between zero and one. Zero indicates it blocks the information entirely, whereas values of one allow all the information to pass.
  (24) $$\begin{array}{l} i^{t}=\sigma \left(W^{\left(ix\right)}x^{\left(t\right)}+U^{\left(ih\right)}h^{\left(t-1\right)}+b^{i}\right) \end{array}$$
- Cell input layer: The cell input has a similar flow as the input gate, receiving *h*^(*t*−1)^ and *x*^(*t*)^ as input. However, a *tanh* activation is used to squish input values to a range between -1 and 1 (denoted by *l*^*t*^ in Equation 25).
  (25) $$\begin{array}{l} l^{t}=\text{tanh}\left(W^{\left(lx\right)}x^{\left(t\right)}+U^{\left(lh\right)}h^{\left(t-1\right)}+b^{l}\right) \end{array}$$
- Forget gate: A unit with a sigmoidal function determines which information from previous steps of the cell should be memorized or forgotten. The forget gate *f*^*t*^ assumes values between zero and one based on the input, *h*^(*t*−1)^ and *x*^(*t*)^. In the next step, *f*^*t*^ is given by a Hadamard product with an old cell state *c*^*t*−1^ to update to a new cell state *c*^*t*^ (Equation 26). In this case, a value of zero means the gate is closed, so it will completely forget the information of the old cell state *c*^*t*−1^, whereas values of one will make all information memorable. Therefore, a forget gate has the right to reset the cell state if the old information is considered meaningless.
  (26) $$\begin{array}{l} f^{t}=\sigma \left(W^{\left(fx\right)}x^{\left(t\right)}+U^{\left(fh\right)}h^{\left(t-1\right)}+b^{f}\right) \end{array}$$
- Cell state: A cell state stores the memory of a cell over a longer time period (Ming et al., 2017). Each cell has a recurrently self-connected linear unit which is called Constant Error Carousel (CEC) (Hochreiter and Schmidhuber, 1997). The CEC mechanism ensures that a LSTM network does not suffer from the vanishing or exploding gradient problem (Elsayed et al., 2018). The CEC is regulated by a forget gate and it can also be reset by the forget gate. At time *t*, the current cell state *c*^*t*^ is updated by the previous cell state *c*^*t*−1^ controlled by the forget gate and the product of the current input and the cell input, i.e., (*i*^*t*^∘*l*^*t*^). Overall, Equation (27) describes the combined update of a cell state,
  (27) $$\begin{array}{l} c^{t}=f^{t}\circ c^{t-1}+i^{t}\circ l^{t}. \end{array}$$
- Output gate: A unit with a sigmoidal function can control the flow of information out of the cell. A LSTM uses the values of the output gate at time *t* (denoted by *o*^*t*^) to control the current cell state *c*^*t*^ activated by a *tanh* function, to obtain the final output vector *h*^(*t*)^,
  (28) $$\begin{array}{l} o^{t}=\sigma \left(W^{\left(ox\right)}x^{\left(t\right)}+U^{\left(oh\right)}h^{\left(t-1\right)}+b^{o}\right), \end{array}$$
  (29) $$\begin{array}{l} h^{t}=o^{t}\circ \text{tanh}\left(c^{t}\right). \end{array}$$

### 8.2. Peephole LSTM

**Figure 15:**
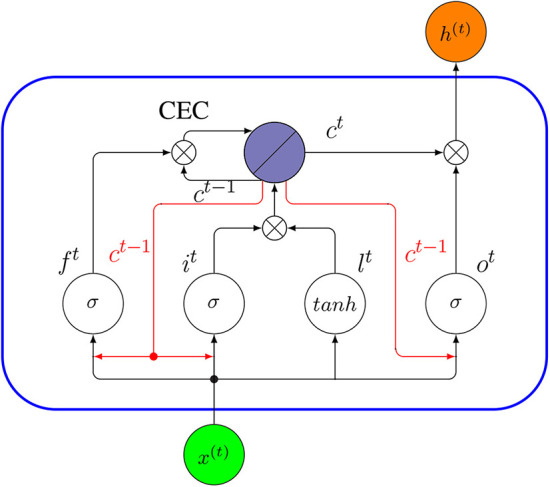
Internal connectivity of a Peephole LSTM unit (blue rectangle). Here *x*^(*t*)^ is the input to the cell at time *t*, and *h*^(*t*)^ is its output. The red arrows are the new peephole connections added, compared to the standard LSTM in Figure 14.

A Peephole LSTM is a variant of a LSTM proposed by Gers and Schmidhuber (2000). In contrast to a standard LSTM discussed above, a Peephole LSTM uses the cell state *c*, instead of *h* for regulating the forget gate, input gate and output gate. In Figure 15, we show the internal connectivity of a Peephole LSTM unit whereas the red arrows represent the new peephole connections.

The key difference between a Peephole LSTM and a standard LSTM is that the forget gate *f*^*t*^, input gate *i*^*t*^ and output gate *o*^*t*^ do not use *h*^(*t*−1)^ as input. Instead, these gates use the cell state *c*^*t*−1^. In order to understand the base idea behind a Peephole LSTM, let us assume the output gate *o*^*t*−1^ in a traditional LSTM network is closed. Then the output of the network *h*^(*t*−1)^ at time (*t*− 1) will be 0, according to Equation (29), and in the next time step *t*, the regulating mechanism of all three gates will only depend on the network input *x*^(*t*−1)^. Therefore, the historical information will be lost completely. A Peephole LSTM avoids this problem by using a cell state instead of output *h* to control the gates. The following equations describe a Peephole LSTM formally.

(30) $$\begin{array}{l} i^{t}=\sigma \left(W^{\left(ix\right)}x^{\left(t\right)}+U^{\left(ic\right)}c^{t-1}+b^{i}\right) \end{array}$$

(31) $$\begin{array}{l} l^{t}=\text{tanh}\left(W^{\left(lx\right)}x^{\left(t\right)}+b^{l}\right) \end{array}$$

(32) $$\begin{array}{l} f^{t}=\sigma \left(W^{\left(fx\right)}x^{\left(t\right)}+U^{\left(fc\right)}c^{t-1}+b^{f}\right) \end{array}$$

(33) $$\begin{array}{l} o^{t}=\sigma \left(W^{\left(ox\right)}x^{\left(t\right)}+U^{\left(oc\right)}c^{t-1}+b^{o}\right) \end{array}$$

(34) $$\begin{array}{l} c^{t}=f^{t}\circ c^{t-1}+i^{t}\circ l^{t} \end{array}$$

(35) $$\begin{array}{l} h^{t}=o^{t}\circ c^{t} \end{array}$$

Aside from these main forms of LSTMs described above, there are further variants. For instance, a Bidirectional LSTM Network (BLSTM) has been introduced by (Graves and Schmidhuber, 2005), which can access long-range context in both input directions. Furthermore, in 2014, the concept of “Gated Recurrent Unit” was proposed, which is viewed as a simplified version of LSTM (Cho et al., 2014) and in 2015, Wai-kin Wong and Wang-chun Woo introduced a Convolutional LSTM Network (ConvLSTM) for precipitation nowcasting (Xingjian et al., 2015). There are further variants of LSTM networks; however, most of them are designed for specific application domains without clear performance advantage.

### 8.3. Applications

**Figure 16:**
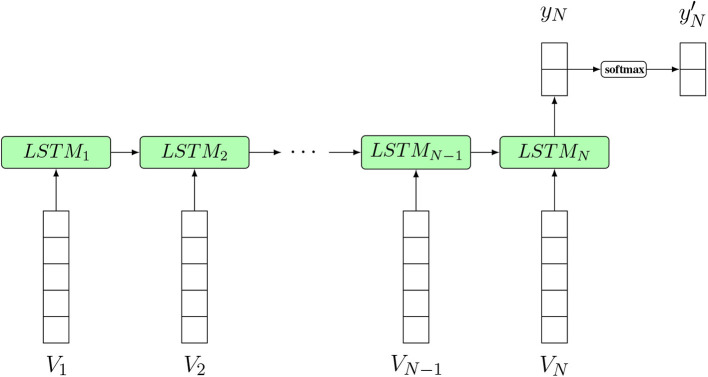
An LSTM classifier model for text classification. *N* is the sequence length of the input text (the number of words). Input from *V*_1_ to *V*_*N*_ is a sequence of word embedding vectors used as input to the model at different time steps. $y_{N}^{\prime }$ is the final prediction result.

LSTMs have a wide range of applications in text generation, text classification, language translation or image captioning (Hwang and Sung, 2015; Vinyals et al., 2015). In Figure 16, an LSTM classifier model for text classification is shown. In this figure, the input of the LSTM structure at each time step is a word embedding vector *V*_*i*_, which is a common choice for text classification problems. A word embedding technique maps the words or phrases in the vocabulary to vectors consisting of real numbers. Some common word embedding techniques include word2vec, GloVe, FastText, etc. Zhou (2019). The output *y*_*N*_ is the corresponding output at the *Nth* time step and $y_{N}^{\prime }$ is the final output after softmax activation of *y*_*N*_, which will determine the classification of the input text.

## 9. Discussion

### 9.1. General Characteristics of Deep Learning

A property common to all deep learning models is that they perform so-called representation learning. Sometimes this is also called feature learning. This denotes a model that learns new and better representations compared to the raw data. Importantly, deep learning models do not learn the final representation within one step but multiple ones corresponding to multi-level representation transformations between the hidden layers (LeCun et al., 2015).

Another common property of deep learning models is that the subsequent transformations between layers are non-linear (see Figure 3). This increases the expressive power of the model (Duda et al., 2000). Furthermore, individual representations are not designed manually, but learned via training data (LeCun et al., 2015). This makes deep learning models very flexible.

### 9.2. Differences Between Models

Currently, CNNs are the dominating deep learning models for computer vision tasks (LeCun et al., 2015). They are effective when the data consist of arrays where nearby values in an array are correlated with each other, e.g., as is the case for images, videos, and sound data. A convolutional layer can easily process high-dimensional input by using the local connectivity and shared weights, while a pooling layer can down-sample the input without losing essential information. Each convolutional layer is capable of converting the input image into groups of more abstract features using different kernels; therefore, by stacking multiple convolution layers, the network is able to transform the input image to a representation that captures essential patterns from the input, thus making precise predictions.

However, also in other areas, CNNs have shown very competitive results compared to other deep learning architectures, e.g., in natural language processing (Kim, 2014; Yang et al., 2020). Specifically, CNNs can be good at extracting local information from text and exploring meaningful semantic and syntactic meanings between phrases and words. Also, the natural composition of text data can be easily handled by a CNN architecture. Hence, CNNs show very strong potential in performing classification tasks where successful predictions heavily rely on extracting key information from input text (Yin et al., 2017).

The classical network architecture is fully connected and feedforward corresponding to a D-FFNN. Interestingly, in (Mayr et al., 2016), it has been shown that a D-FFNN outperformed other methods for predicting the toxicity of drugs. Also for drug target predictions, a D-FFNN has been shown to be superior compared to other methods (Mayr et al., 2018). This shows that even such an architecture can be successfully used in modern applications.

Commonly, RNNs are used for problems with sequential data, such as speech and language processing or modeling (Sundermeyer et al., 2012; Graves et al., 2013; Luong and Manning, 2015). While DBNs and CNNs are feedforward networks, connections in RNNs can form cycles. This allows the modeling of dynamical changes over time (LeCun et al., 2015).

A problem with finding the right application for a deep learning model is that their application domains are not mutually exclusive from each other. Instead, as the discussion above shows, there is a considerable overlap and the best model can in many cases only be found by conducting a comparative study. In Table 3, we show several examples of different applications involving images, audio, text, and genomics data.

**Table 3:** Overview of applications of deep learning methods.

**Description**	**DL type**	**Application**	**References**
Dermatologist-level classification of skin cancer with deep neural networks	CNN	Images	Esteva et al., 2017
Deep learning for lung cancer prognostication: a retrospective multi-cohort radiomics study	CNN	Images	Hosny et al., 2018
Character-level convolutional networks for text classification	CNN	Text	Zhang X. et al., 2015
Recurrent convolutional neural networks for text classification	CNN	Text	Lai et al., 2015
Comparing deep belief networks with support vector machines for classifying gene expression data from complex disorders	DBN	Genomics	Smolander et al., 2019a
Unsupervised feature learning for audio classification using convolutional deep belief networks	C-DBN	Audio	Lee et al., 2009
Acoustic modeling using deep belief networks	DBN	Audio	Mohamed et al., 2011
Jiang, M., et al. Text classification based on deep belief network and softmax regression	DBN	Text	Jiang et al., 2018
Autoencoder for words	AE	Text	Liou et al., 2014
Deep neural networks for learning graph representations	AE	Text	Cao et al., 2016
Stacked denoising autoencoders: learning useful representations in a deep network with a local denoising criterion	SD-AE	Images	Vincent et al., 2010
DeepCare: a deep dynamic memory model for predictive medicine	LSTM	Text	Pham et al., 2016
Framewise phoneme classification with bidirectional LSTM and other neural network architectures	B-LSTM	Audio	Graves and Schmidhuber, 2005
Deep sentence embedding using long short-term memory networks: analysis and application to information retrieval	LSTM	Text	Palangi et al., 2016
Drug-drug interaction extraction from biomedical texts using long short-term memory network	LSTM	Text	Sahu and Anand, 2018

### 9.3. Interpretable Models vs. Black-Box Models

Any model in data science can be categorized either as an *inferential model* or a *prediction model* (Breiman, 2001; Shmueli, 2010). An inferential model does not only make predictions but provides also an interpretable structure. Hence, it is a model of the prediction process itself, e.g., a causal model. In contrast, a prediction model is merely a black-box model for making predictions.

The models discussed in this review neither aim at providing physiological models of biological neurons nor offer an interpretable structure. Instead, they are prediction models. An example for a biologically motivated learning rule for neural networks is the Hebbian learning rule (Hebb, 1949). Hebbian learning is a form of unsupervised learning of neural networks that does not use global information about the error as backpropagation. Instead, only local information is used from adjacent neurons. There are many extensions of Hebb's basic learning rule that have been introduced based on new biological insights (see e.g., Emmert-Streib, 2006).

Recently, there is great interest in interpretable or explainable AI (XAI) (Biran and Cotton, 2017; Doshi-Velez and Kim, 2017). Especially in the clinical and medical area, one would like to have understandable decisions of statistical prediction models because patients are affected (Holzinger et al., 2017). The field is still in its infancy, but if meaningful interpretations of general deep learning models could be found this would certainly revolutionize the field.

As a note, we would like to add that the distinction between an explainable AI model and a non-explainable model is not well-defined. For instance, the sparse coding model by Olshausen and Field (1997) was shown to be similar to the coding of images in the human visual cortex (Tosic and Frossard, 2011) and an application of this model can be found in Charles et al. (2011), where an unsupervised learning approach was used to learn an optimal sparse coding dictionary for the classification of high spectral imagery (HIS) data. Some may consider this model as an XAI model because of the similarity to the working mechanism of the human cortex, whereas others may question this explanation.

### 9.4. Big Data vs. Small Data

In statistics, the field of experimental design is concerned with assessing if the available sample sizes are sufficient to conduct a particular analysis (for a practical example see Stupnikov et al., 2016). In contrast, for all methods discussed in this paper, we assumed that we are in the big data domain implying sufficient samples. This corresponds to the ideal case. However, we would like to point out that for practical applications, one needs to assess this situation case-by-case to ensure the available data (respectively the sample sizes) are sufficient to use deep learning models. Unfortunately, this issue is not well-represented in the current literature. As a *rule-of-thumb*, deep learning models usually perform well for tens of thousands of samples but it is largely unclear how they perform in a small data setting. This leaves it to the user to estimate learning curves of the generalization error for a given model to avoid spurious results (Emmert-Streib and Dehmer, 2019b).

As an example to demonstrate this problem, we conducted an analysis to explore the influence of the sample size on the accuracy of the classification of the EMNIST data. EMNIST (Extended MNIST) (Cohen et al., 2017) consists of 280, 000 handwritten characters (240, 000 training samples and 40, 000 test samples) for 10 balanced classes (0–9). We used a multilayered Long Short-Term Memory (LSTM) model for the 10-class handwritten digit classification task. The model we used is a four-layer network (three hidden layers and one fully connected layer), and each hidden layer contains 200 neurons. For this analysis, we set the batch size to 100 and the training samples were randomly drawn if the number of training samples was < 240, 000 (subsampling).

**Figure 17:**
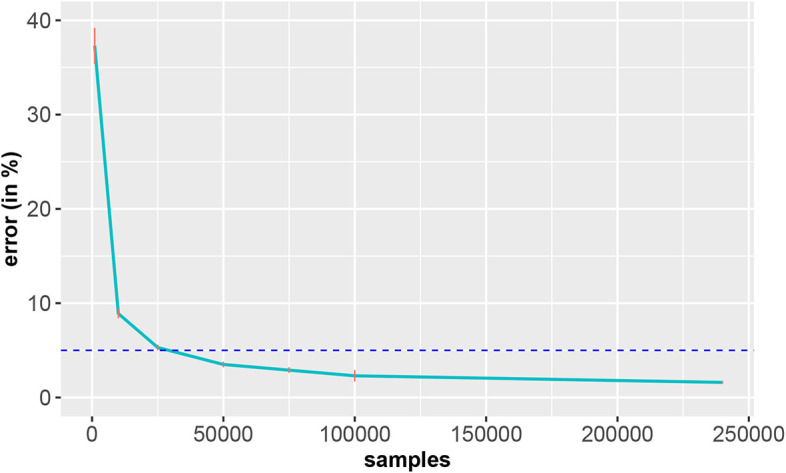
Classification error of the EMNIST data in dependence on the number of training samples. The standard errors are shown in red and the horizontal dashed line corresponds to an error of 5% (reference). The results are averaged over 10 independent runs.

From the results in Figure 17, one can see that thousands of training samples are needed to achieve a classification error below 5% (blue dashed line). Specifically, more than 25, 000 training samples are needed. Given the relative simplicity of the problem—classification of ten digits, compared to classification or diagnosis of cancer patients—the severity of this issue should become clear. Also, these results show that a deep learning model cannot do miracles. If the number of samples is too small, the method breaks down. Hence, the combination of a model and data is crucial for solving a task.

### 9.5. Data Types

A related problem to the sample size issue discussed above is the type of data. Examples for different data types are text data, image data, audio data, network data or measurement/sensor data (for instance from genomics) to name just a few. One can further subdivide these data according to the application domain from which these originate, e.g., text data from medical publications, text data from social media or text data from novels. Considering such categorizations, it becomes clear that the information content of 'one sample' does not have the same meaning for each data type and for each application domain. Hence, the assessment of deep learning models needs to be always conducted in a domain specific manner because the transfer of knowledge between such models is not straight forward.

### 9.6. Further Advanced Models

Finally, we would like to emphasize that there are additional but more advanced models of deep learning networks, which are outside the core architectures. For instance, deep learning and reinforcement learning have been combined with each other to form deep reinforcement learning (Mnih et al., 2015; Arulkumaran et al., 2017; Henderson et al., 2018). Such models have found application in problems from robotics, games and healthcare.

Another example for an advanced model is a graph CNN, which is particularly suitable when data have the form of graphs (Henaff et al., 2015; Wu et al., 2019). Such models have been used in natural language processing, recommender systems, genomics and chemistry (Li et al., 2018; Yao et al., 2019).

Lastly, a further advanced model is a Variational Autoencoder (VAE) (An and Cho, 2015; Doersch, 2016). Put simply, a VAR is a regularized Autoencoder that uses a distribution over the latent spaces as encoding for the input, instead of a single point. The major application of VAE is as a generative model for generating similar data in an unsupervised manner, e.g., for image or text generation.

## 10. Conclusion

In this paper, we provided an introductory review for deep learning models including Deep Feedforward Neural Networks, (D-FFNN), Convolutional Neural Networks (CNNs), Deep Belief Networks (DBNs), Autoencoders (AE) and Long Short-Term Memory networks (LSTMs). These models can be considered the core architectures that currently dominate deep learning. In addition, we discussed related concepts needed for a technical understanding of these models, e.g., Restricted Boltzmann Machines and resilient backpropagation. Given the flexibility of network architectures allowing a “*Lego-like*” construction of new models, an unlimited number of neural network models can be constructed by utilizing elements of the core architectural building blocks discussed in this review. Hence, a basic understanding of these elements is key to be equipped for future developments in AI.

## Author Contributions

FE-S conceived the study. All authors contributed to all aspects of the preparation and the writing of the manuscript.

### Conflict of Interest

The authors declare that the research was conducted in the absence of any commercial or financial relationships that could be construed as a potential conflict of interest.

## References

[B1] Alipanahi B. Delong A. Weirauch M. T. Frey B. J. (2015). Predicting the sequence specificities of DNA-and RNA-binding proteins by deep learning. Nat. Biotechnol. 33, 831–838. 10.1038/nbt.3300 26213851

[B2] An J. Cho S. (2015). Variational Autoencoder Based Anomaly Detection Using Reconstruction Probability. Special Lecture on IE 2.

[B3] Arulkumaran K. Deisenroth M. P. Brundage M. Bharath A. A. (2017). Deep reinforcement learning: a brief survey. IEEE Signal Process. Mag. 34, 26–38. 10.1109/MSP.2017.2743240

[B4] Bergmeir C. Benítez J. M. (2012). Neural networks in R using the stuttgart neural network simulator: RSNNS. J. Stat. Softw. 46, 1–26. 10.18637/jss.v046.i0722837731

[B5] Biran O. Cotton C. (2017). Explanation and justification in machine learning: a survey, in IJCAI-17 Workshop on Explainable AI (XAI). Vol. 8, 1.

[B6] Bottou L. (2010). Large-scale machine learning with stochastic gradient descent, in Proceedings of COMPSTAT'2010 (Springer), 177–186.

[B7] Breiman L. (2001). Statistical modeling: the two cultures (with comments and a rejoinder by the author). Stat. Sci. 16, 199–231. 10.1214/ss/1009213726

[B8] Cao C. Liu F. Tan H. Song D. Shu W. Li W.. (2018). Deep learning and its applications in biomedicine. Genomics Proteomics Bioinform. 16, 17–32. 10.1016/j.gpb.2017.07.003 29522900 PMC6000200

[B9] Cao S. Lu W. Xu Q. (2016). Deep neural networks for learning graph representations, in Thirtieth AAAI Conference on Artificial Intelligence.

[B10] Carreira-Perpinan M. A. Hinton G. E. (2005). On contrastive divergence learning, in Proceedings of the Tenth International Workshop on Artificial Intelligence and Statistics (Citeseer), 33–40.

[B11] Charles A. S. Olshausen B. A. Rozell C. J. (2011). Learning sparse codes for hyperspectral imagery. IEEE J. Select. Top. Signal Process. 5, 963–978. 10.1109/JSTSP.2011.2149497

[B12] Chen T. Li M. Li Y. Lin M. Wang N. Wang M. (2015). Mxnet: a flexible and efficient machine learning library for heterogeneous distributed systems.

[B13] Chimera (2019). Pydbm. arXiv:1512.01274.

[B14] Cho K. Van Merriënboer B. Gulcehre C. Bahdanau D. Bougares F. Schwenk H. (2014). Learning phrase representations using rnn encoder-decoder for statistical machine translation. arXiv [Preprint]. arXiv:1406.1078. 10.3115/v1/D14-1179

[B15] Chollet F. (2015). Keras. Available online at: https://github.com/fchollet/keras

[B16] Cohen G. Afshar S. Tapson J. van Schaik A. (2017). Emnist: an extension of mnist to handwritten letters. arXiv[Preprint]. arXiv:1702.05373 10.1109/IJCNN.2017.7966217

[B17] Dai J. Wang Y. Qiu X. Ding D. Zhang Y. Wang Y. (2018). BigDL: a distributed deep learning framework for big data. arXiv:1804.05839.

[B18] [Dataset] Abadi M. Agarwal A. Barham P. Brevdo E. Chen Z. Citro C. (2016). Tensorflow: Large-scale machine learning on heterogeneous distributed systems. arXiv:1603.04467.

[B19] [Dataset] Bondarenko Y. (2017). Boltzman-Machines.

[B20] [Dataset] Candel A. Pramar V. LeDell E. Arora A. (2015). Deep Learning With H2O.

[B21] [Dataset] Dieleman S. Schlüter J. Raffel C. Olson E. Sonderby S. K. Nouri D. (2015). Lasagne: First Release.

[B22] [Dataset] Howard J Gugger S (2018). fastai: A Layered API for Deep Learning. arXiv:2002.04688.

[B23] Deng J. Zhang Z. Marchi E. Schuller B. (2013). Sparse autoencoder-based feature transfer learning for speech emotion recognition, in 2013 Humaine Association Conference on Affective Computing and Intelligent Interaction (IEEE), 511–516.

[B24] Dixon M. Klabjan D. Wei L. (2017). Ostsc: over sampling for time series classification in R.

[B25] Doersch C. (2016). Tutorial on variational autoencoders. arXiv [Preprint]. arXiv:1606.05908.

[B26] Donahue J. Jia Y. Vinyals O. Hoffman J. Zhang N. Tzeng E. (2014). Decaf: a deep convolutional activation feature for generic visual recognition, in International Conference on Machine Learning, 647–655.

[B27] Doshi-Velez F. Kim B. (2017). Towards a rigorous science of interpretable machine learning. arXiv [Preprint]. arXiv:1702.08608.

[B28] Duda R. O. Hart P. E. Stork D. G. (2000). Pattern Classification. 2nd Edn. Wiley.

[B29] Elsayed N. Maida A. S. Bayoumi M. (2018). Reduced-gate convolutional LSTM using predictive coding for spatiotemporal prediction. arXiv [Preprint]. arXiv:1810.07251.

[B30] Emmert-Streib F. (2006). A heterosynaptic learning rule for neural networks. Int. J. Mod. Phys. C 17, 1501–1520. 10.1142/S0129183106009916

[B31] Emmert-Streib F. Dehmer M. (2019a). Defining data science by a data-driven quantification of the community. Mach. Learn. Knowl. Extract. 1, 235–251. 10.3390/make1010015

[B32] Emmert-Streib F. Dehmer M. (2019b). Evaluation of regression models: model assessment, model selection and generalization error. Mach. Learn. Knowl. Extract. 1, 521–551. 10.3390/make1010032

[B33] Enarvi S. Kurimo M. (2016). TheanoLM–an extensible toolkit for neural network language modeling. Proc. Interspeech 3052–3056 10.21437/Interspeech.2016-618

[B34] Esteva A. Kuprel B. Novoa R. A. Ko J. Swetter S. M. Blau H. M.. (2017). Dermatologist-level classification of skin cancer with deep neural networks. Nature 542:115. 10.1038/nature21056 28117445 PMC8382232

[B35] Fischer A. Igel C. (2012). An introduction to restricted boltzmann machines, in Progress in Pattern Recognition, Image Analysis, Computer Vision, and Applications (Springer), 14–36.

[B36] Fodor J. A. Pylyshyn Z. W. (1988). Connectionism and cognitive architecture: a critical analysis. Cognition 28, 3–71. 2450716 10.1016/0010-0277(88)90031-5

[B37] Fukushima K. (1980). Neocognitron: A self-organizing neural network model for a mechanism of pattern recognition unaffected by shift in position. Biol. Cybernet. 36, 193–202. 7370364 10.1007/BF00344251

[B38] Fukushima K. (2013). Training multi-layered neural network neocognitron. Neural Netw. 40, 18–31. 10.1016/j.neunet.2013.01.001 23380595

[B39] Gers F. A. Schmidhuber J. (2000). Recurrent nets that time and count, in Proceedings of the IEEE-INNS-ENNS International Joint Conference on Neural Networks. IJCNN 2000. Neural Computing: New Challenges and Perspectives for the New Millennium (IEEE), Vol. 3, 189–194.

[B40] Gers F. A. Schmidhuber J. Cummins F. (1999). Learning to forget: continual prediction with LSTM. Neural Comput. 12, 2451–2471. 10.1162/089976600300015015 11032042

[B41] Gers F. A. Schraudolph N. N. Schmidhuber J. (2002). Learning precise timing with lstm recurrent networks. J. Mach. Learn. Res. 3, 115–143. Available online at: http://www.jmlr.org/papers/v3/gers02a.html

[B42] Goodfellow I. Bengio Y. Courville A. (2016). Deep Learning. MIT Press.

[B43] Goodfellow I. Pouget-Abadie J. Mirza M. Xu B. Warde-Farley D. Ozair S. (2014). Generative adversarial nets, in Advances in Neural Information Processing Systems, 2672–2680.

[B44] Goodfellow I. J. Warde-Farley D. Lamblin P. Dumoulin V. Mirza M. Pascanu R. (2013). Pylearn2: a machine learning research library. arXiv:1308.4214.

[B45] Graves A. (2013). Generating sequences with recurrent neural networks. arXiv [Preprint]. arXiv:1308.0850.

[B46] Graves A. Mohamed A. Hinton G. E. (2013). Speech recognition with deep recurrent neural networks, in 2013 IEEE International Conference on Acoustics, Speech and Signal Processing (ICASSP).

[B47] Graves A. Schmidhuber J. (2005). Framewise phoneme classification with bidirectional LSTM and other neural network architectures. Neural Netw. 18, 602–610. 10.1016/j.neunet.2005.06.042 16112549

[B48] Hastie T. J. Tibshirani R. J. Friedman J. H. (2009). The Elements of Statistical Learning: Data Mining, Inference, and Prediction. Springer Series in Statistics. Springer.

[B49] Hayter H. O. (2012). Probability and Statistics for Engineers and Scientists. 4th Edn. Duxbury Press).

[B50] He K. Zhang X. Ren S. Sun J. (2016). Deep residual learning for image recognition, in Proceedings of the IEEE Conference on Computer Vision and Pattern Recognition, 770–778.

[B51] Hebb D. (1949). The Organization of Behavior. New York, NY: Wiley.

[B52] Henaff M. Bruna J. LeCun Y. (2015). Deep convolutional networks on graph-structured data. arXiv [Preprint]. arXiv:1506.05163.

[B53] Henderson P. Islam R. Bachman P. Pineau J. Precup D. Meger D. (2018). Deep reinforcement learning that matters, in Thirty-Second AAAI Conference on Artificial Intelligence.

[B54] Hertz J. Krogh A. Palmer R. (1991). Introduction to the Theory of Neural Compuation. Addison-Wesley.

[B55] Hinton G. E. (2012). Neural Networks: Tricks of the Trade. 2nd Edn. Chapter. A Practical Guide to Training Restricted Boltzmann Machines. Berlin; Heidelberg: Springer Berlin Heidelberg, 599–619.

[B56] Hinton G. E. Osindero S. Teh Y.-W. (2006). A fast learning algorithm for deep belief nets. Neural Comput. 18, 1527–1554. 10.1162/neco.2006.18.7.1527 16764513

[B57] Hinton G. E. Salakhutdinov R. R. (2006). Reducing the dimensionality of data with neural networks. Science 313, 504–507. 10.1126/science.1127647 16873662

[B58] Hinton G. E. Sejnowski T. J. (1983). Optimal perceptual inference, in Proceedings of the IEEE conference on Computer Vision and Pattern Recognition (Citeseer), 448–453.

[B59] Hochreiter S. (1991). Untersuchungen zu Dynamischen Neuronalen Netzen. Diploma, Technische Universität München 91.

[B60] Hochreiter S. (1998). The vanishing gradient problem during learning recurrent neural nets and problem solutions. Int. J. Uncertainty Fuzziness Knowl. Based Syst. 6, 107–116.

[B61] Hochreiter S. Schmidhuber J. (1997). Long short-term memory. Neural Comput. 9, 1735–1780. 9377276 10.1162/neco.1997.9.8.1735

[B62] Holzinger A. Biemann C. Pattichis C. S. Kell D. B. (2017). What do we need to build explainable AI systems for the medical domain? arXiv [Preprint]. arXiv:1712.09923.

[B63] Hopfield J. (1982). Neural networks and physical systems with emergent collective computational abilities. Proc. Natl. Acad. Sci. U.S.A. 79, 2554–2558. 6953413 10.1073/pnas.79.8.2554 PMC346238

[B64] Hoppensteadt F. C. Izhikevich E. M. (1999). Oscillatory neurocomputers with dynamic connectivity. Phys. Rev. Lett. 82:2983.

[B65] Hornik K. (1991). Approximation capabilities of multilayer feedforward networks. Neural Netw. 4, 251–257.

[B66] Hosny A. Parmar C. Coroller T. P. Grossmann P. Zeleznik R. Kumar A.. (2018). Deep learning for lung cancer prognostication: a retrospective multi-cohort radiomics study. PLoS Med. 15:e1002711. 10.1371/journal.pmed.1002711 30500819 PMC6269088

[B67] Hwang K. Sung W. (2015). Single stream parallelization of generalized LSTM-like rnns on a GPU, in 2015 IEEE International Conference on Acoustics, Speech and Signal Processing (ICASSP) (IEEE), 1047–1051.

[B68] Igel C. Hüsken M. (2000). Improving the RPROP learning algorithm, in Proceedings of the Second International ICSC Symposium on Neural Computation (NC 2000), Vol. 2000 (Citeseer), 115–121.

[B69] Ivakhnenko A. G. (1968). The group method of data of handling; a rival of the method of stochastic approximation. Soviet Autom. Control 13, 43–55.

[B70] Ivakhnenko A. G. (1971). Polynomial theory of complex systems. IEEE Trans. Syst. Man Cybernet. SMC-1, 364–378.

[B71] Jia Y. Shelhamer E. Donahue J. Karayev S. Long J. Girshick R. (2014). Caffe: convolutional architecture for fast feature embedding, in Proceedings of the 22Nd ACM International Conference on Multimedia, MM '14 (New York, NY: ACM), 675–678.

[B72] Jiang M. Liang Y. Feng X. Fan X. Pei Z. Xue Y. (2018). Text classification based on deep belief network and softmax regression. Neural Comput. Appl. 29, 61–70. 10.1007/s00521-016-2401-x

[B73] Kim Y. (2014). Convolutional neural networks for sentence classification. arXiv [Preprint]. arXiv:1408.5882 10.3115/v1/D14-1181

[B74] Kou Q. Sugomori Y. (2014). Rcppdl.

[B75] Kraemer G. Reichstein M. D. M. M (2018). dimRed and coRanking—unifying dimensionality reduction in R. R J. 10, 342–358. 10.32614/RJ-2018-039

[B76] Krizhevsky A. Sutskever I. Hinton G. E. (2012a). ImageNet Classification with Deep Convolutional Neural Networks. Curran Associates, Inc.

[B77] Krizhevsky A. Sutskever I. Hinton G. E. (2012b). Imagenet classification with deep convolutional neural networks, in Advances in Neural Information Processing Systems, 1097–1105.

[B78] Lai S. Xu L. Liu K. Zhao J. (2015). Recurrent convolutional neural networks for text classification, in Twenty-Ninth AAAI Conference on Artificial Intelligence.

[B79] Lawrence S. Giles C. L. Tsoi A. C. Back A. D. (1997). Face recognition: a convolutional neural-network approach. IEEE Trans. Neural Netw. 8, 98–113. 18255614 10.1109/72.554195

[B80] Le Cun Y. (1989). Generalization and Network Design Strategies. Technical Report CRG-TR-89-4, Connectionism in Perspective. University of Toronto Connectionist Research Group, Toronto, ON.

[B81] LeCun Y. Bengio Y. Hinton G. (2015). Deep learning. Nature 521:436. 26017442 10.1038/nature14539

[B82] LeCun Y. Boser B. Denker J. S. Henderson D. Howard R. E. Hubbard W. (1989). Backpropagation applied to handwritten zip code recognition. Neural Comput. 1, 541–551.

[B83] Lee H. Pham P. Largman Y. Ng A. Y. (2009). Unsupervised feature learning for audio classification using convolutional deep belief networks, in Advances in Neural Information Processing Systems, 1096–1104.

[B84] Leung M. K. K. Xiong H. Y. Lee L. J. Frey B. J. (2014). Deep learning of the tissue-regulated splicing code. Bioinformatics 30, 121–129. 10.1093/bioinformatics/btu277 24931975 PMC4058935

[B85] Li R. Wang S. Zhu F. Huang J. (2018). Adaptive graph convolutional neural networks, in Thirty-Second AAAI Conference on Artificial Intelligence.

[B86] Lin M. Chen Q. Yan S. (2013). Network in network. arXiv [Preprint]. arXiv:1312.4400.

[B87] Linnainmaa S. (1976). Taylor expansion of the accumulated rounding error. BIT Numer. Math. 16, 146–160.

[B88] Liou C.-Y. Cheng W.-C. Liou J.-W. Liou D.-R. (2014). Autoencoder for words. Neurocomputing 139, 84–96. 10.1016/j.neucom.2013.09.055

[B89] Lipton Z. C. Berkowitz J. Elkan C. (2015). A critical review of recurrent neural networks for sequence learning. arXiv [Preprint]. arXiv:1506.00019.

[B90] Lu Z. Pu H. Wang F. Hu Z. Wang L. (2017). The expressive power of neural networks: a view from the width, in Advances in Neural Information Processing Systems, 6231–6239.

[B91] Luong M.-T. Manning C. D. (2015). Stanford neural machine translation systems for spoken language domains, in Proceedings of the International Workshop on Spoken Language Translation, 76–79.

[B92] Mayr A. Klambauer G. Unterthiner T. Hochreiter S. (2016). Deeptox: toxicity prediction using deep learning. Front. Environ. Sci. 3:80 10.3389/fenvs.2015.00080

[B93] Mayr A. Klambauer G. Unterthiner T. Steijaert M. Wegner J. K. Ceulemans H.. (2018). Large-scale comparison of machine learning methods for drug target prediction on chembl. Chem. Sci. 9, 5441–5451. 10.1039/C8SC00148K 30155234 PMC6011237

[B94] McCulloch W. Pitts W. (1943). A logical calculus of the ideas immanent in nervous activity. Bull. Math. Biophys. 5, 115–133. 2185863

[B95] Ming Y. Cao S. Zhang R. Li Z. Chen Y. Song Y. (2017). Understanding hidden memories of recurrent neural networks, in 2017 IEEE Conference on Visual Analytics Science and Technology (VAST) (IEEE), 13–24.

[B96] Minsky M. Papert S. (1969). Perceptrons. MIT Press.

[B97] Mnih V. Kavukcuoglu K. Silver D. Rusu A. A. Veness J. Bellemare M. G.. (2015). Human-level control through deep reinforcement learning. Nature 518:529. 10.1038/nature14236 25719670

[B98] Mohamed A.-R. Dahl G. E. Hinton G. (2011). Acoustic modeling using deep belief networks. IEEE Trans. Audio Speech Lang. Process. 20, 14–22. 10.1109/TASL.2011.2109382

[B99] Nair V. Hinton G. E. (2010). Rectified linear units improve restricted boltzmann machines, in Proceedings of the 27th International Conference on Machine Learning (ICML-10), 807–814.

[B100] Nielsen M. A. (2015). Neural Networks and Deep Learning. Determination Press.

[B101] Olshausen B. A. Field D. J. (1997). Sparse coding with an overcomplete basis set: a strategy employed by v1? Vision Res. 37, 3311–3325. 9425546 10.1016/s0042-6989(97)00169-7

[B102] Palangi H. Deng L. Shen Y. Gao J. He X. Chen J. (2016). Deep sentence embedding using long short-term memory networks: Analysis and application to information retrieval. IEEE/ACM Trans. Audio Speech Lang. Process. 24, 694–707. 10.1109/TASLP.2016.2520371

[B103] Paszke A. Gross S. Chintala S. Chanan G. Yang E. DeVito Z. (2017). Automatic differentiation in pytorch. Available online at: https://www.semanticscholar.org/paper/Automatic-differentiation-in-PyTorch-Paszke-Gross/b36a5bb1707bb9c70025294b3a310138aae8327a

[B104] Pedregosa F. Varoquaux G. Gramfort A. Michel V. Thirion B. Grisel O. (2011). Scikit-learn: machine learning in Python. J. Mach. Learn. Res. 12, 2825–2830. Available online at: http://www.jmlr.org/papers/v12/pedregosa11a

[B105] Pham T. Tran T. Phung D. Venkatesh S. (2016). Deepcare: a deep dynamic memory model for predictive medicine, in Pacific-Asia Conference on Knowledge Discovery and Data Mining (Springer), 30–41.

[B106] Pu Y. Gan Z. Henao R. Yuan X. Li C. Stevens A. (2016). Variational autoencoder for deep learning of images, labels and captions, in Advances in Neural Information Processing Systems, 2352–2360.

[B107] Quast B. (2016). RNN: A Recurrent Neural Network in R. Working Papers.

[B108] Rawat W. Wang Z. (2017). Deep convolutional neural networks for image classification: a comprehensive review. Neural Comput. 29, 2352–2449. 10.1162/neco_a_00990 28599112

[B109] Riedmiller M. Braun H. (1993). A direct adaptive method for faster backpropagation learning: the rprop algorithm, in IEEE International Conference on Neural Networks (IEEE), 586–591.

[B110] Rong X. (2014). Deep Learning Toolkit in R.

[B111] Rosenblatt F. (1957). The Perceptron, A Perceiving and Recognizing Automaton Project Para. Cornell Aeronautical Laboratory.

[B112] Rumelhart D. Hinton G. Williams R. (1986). Learning representations by back-propagating errors. Nature 323, 533–536.

[B113] Sahu S. K. Anand A. (2018). Drug-drug interaction extraction from biomedical texts using long short-term memory network. J. Biomed. Inform. 86, 15–24. 10.1016/j.jbi.2018.08.005 30142385

[B114] Salakhutdinov R. Hinton G. E. (2009). Deep boltzmann machines, in International conference on artificial intelligence and statistics, 448–455.

[B115] Sarikaya R. Hinton G. E. Deoras A. (2014). Application of deep belief networks for natural language understanding. IEEE/ACM Trans. Audio Speech Lang. Process. 22, 778–784. 10.1109/TASLP.2014.2303296

[B116] Scherer D. Müller A. Behnke S. (2010). Evaluation of pooling operations in convolutional architectures for object recognition, in International Conference on Artificial Neural Networks (Springer), 92–101.

[B117] Schmidhuber J. (1992). Learning complex, extended sequences using the principle of history compression. Neural Comput. 4, 234–242.

[B118] Schmidhuber J. (2015). Deep learning in neural networks: an overview. Neural Netw. 61, 85–117. 10.1016/j.neunet.2014.09.003 25462637

[B119] Sejnowski T. J. Rosenberg C. R. (1987). Parallel networks that learn to pronounce english text. Complex Syst. 1, 145–168.

[B120] Shen D. Wu G. Suk H.-I. (2017). Deep learning in medical image analysis. Annu. Rev. Biomed. Eng. 19, 221–248. 10.1146/annurev-bioeng-071516-044442 28301734 PMC5479722

[B121] Shmueli G. (2010). To explain or to predict? Stat. Sci. 25, 289–310. 10.1214/10-STS330

[B122] Simonyan K. Zisserman A. (2014). Very deep convolutional networks for large-scale image recognition. arXiv [Preprint]. arXiv:1409.1556.

[B123] Smolander J. (2016). Deep learning classification methods for complex disorders (Master's thesis), The School of the thesis, Tampere University of Technology, Tampere, Finland. Available online at: https://dspace.cc.tut.fi/dpub/handle/123456789/23845

[B124] Smolander J. Dehmer M. Emmert-Streib F. (2019a). Comparing deep belief networks with support vector machines for classifying gene expression data from complex disorders. FEBS Open Bio 9, 1232–1248. 10.1002/2211-5463.12652 31074948 PMC6609581

[B125] Smolander J. Stupnikov A. Glazko G. Dehmer M. Emmert-Streib F. (2019b). Comparing biological information contained in mRNA and non-coding RNAs for classification of lung cancer patients. BMC Cancer 19:1176. 10.1186/s12885-019-6338-1 31796020 PMC6892207

[B126] Soman K. Muralidharan V. Chakravarthy V. S. (2018). An oscillatory neural autoencoder based on frequency modulation and multiplexing. Front. Comput. Neurosci. 12:52. 10.3389/fncom.2018.00052 30042669 PMC6048285

[B127] Stupnikov A. Tripathi S. de Matos Simoes R. McArt D. Salto-Tellez M. Glazko G.. (2016). samExploreR: exploring reproducibility and robustness of RNA-seq results based on SAM files. Bioinformatics 32, 3345–3347. 10.1093/bioinformatics/btw475 27402900

[B128] Sundermeyer M. Schlüter R. Ney H. (2012). LSTM neural networks for language modeling, in Thirteenth Annual Conference of the International Speech Communication Association.

[B129] Szegedy C. Liu W. Jia Y. Sermanet P. Reed S. Anguelov D. (2015). Going deeper with convolutions, in Proceedings of the IEEE Conference on Computer Vision and Pattern Recognition, 1–9.

[B130] Theano Development Team (2016). Theano: a Python framework for fast computation of mathematical expressions. arXiv [Preprint]. arXiv:abs/1605.02688.

[B131] Tosic I. Frossard P. (2011). Dictionary learning. IEEE Signal Process. Mag. 28, 27–38. 10.1109/TIP.2010.2081679 20889431

[B132] Venkataraman S. Yang Z. Liu D. Liang E. Falaki H. Meng X. (2016). Sparkr: Scaling R programs with spark, in Proceedings of the 2016 International Conference on Management of Data, SIGMOD '16 (New York, NY: ACM), 1099–1104. 10.1145/2882903.2903740

[B133] Vincent P. Larochelle H. Lajoie I. Bengio Y. Manzagol P. -A. (2010). Stacked denoising autoencoders: learning useful representations in a deep network with a local denoising criterion. J. Mach. Learn. Res. 11, 3371–3408. Available online at: http://www.jmlr.org/papers/v11/vincent10a.html

[B134] Vinyals O. Toshev A. Bengio S. Erhan D. (2015). Show and tell: a neural image caption generator, in Proceedings of the IEEE Conference on Computer Vision and Pattern Recognition, 3156–3164.

[B135] Wan L. Zeiler M. Zhang S. Cun Y. L. Fergus R. (2013). Regularization of neural networks using dropconnect, in Proceedings of the 30th International Conference on Machine Learning (ICML-13), 1058–1066.

[B136] Wang D. Terman D. (1995). Locally excitatory globally inhibitory oscillator networks. IEEE Trans. Neural Netw. 6, 283–286. 18263312 10.1109/72.363423

[B137] Wang D. Terman D. (1997). Image segmentation based on oscillatory correlation. Neural Comput. 9, 805–836. 9161023 10.1162/neco.1997.9.4.805

[B138] Wang D. L. Brown G. J. (1999). Separation of speech from interfering sounds based on oscillatory correlation. IEEE Trans. Neural Netw. 10, 684–697. 18252568 10.1109/72.761727

[B139] Wang Y. Huang M. Zhao L. (2016). Attention-based lstm for aspect-level sentiment classification, in Proceedings of the 2016 Conference on Empirical Methods in Natural Language Processing, 606–615.

[B140] Webb A. R. Copsey K. D. (2011). Statistical Pattern Recognition. 3rd Edn. Wiley.

[B141] Werbos P. (1974). Beyond regression: new tools for prediction and analysis in the behavioral sciences (Ph.D. thesis), Harvard University, Harvard, MA, United States.

[B142] Werbos P. J. (1981). Applications of advances in nonlinear sensitivity analysis, in Proceedings of the 10th IFIP Conference, 31.8–4.9, New York, 762–770.

[B143] Widrow B. Hoff M. E. (1960). Adaptive Switching Circuits. Technical Report, Stanford University, California; Stanford Electronics Labs.

[B144] Wu Z. Pan S. Chen F. Long G. Zhang C. Yu P. S. (2019). A comprehensive survey on graph neural networks. arXiv [Preprint]. arXiv:1901.00596. 10.1109/TNNLS.2020.2978386 32217482

[B145] Xingjian S. Chen Z. Wang H. Yeung D.-Y. Wong W.-K. Woo W.-C. (2015). Convolutional lstm network: a machine learning approach for precipitation nowcasting, in Advances in Neural Information Processing Systems, 802–810.

[B146] Yang Z. Dehmer M. Yli-Harja O. Emmert-Streib F. (2020). Combining deep learning with token selection for patient phenotyping from electronic health records. Sci. Rep. 10:1432. 10.1038/s41598-020-58178-1 31996705 PMC6989657

[B147] Yao L. Mao C. Luo Y. (2019). Graph convolutional networks for text classification, in Proceedings of the AAAI Conference on Artificial Intelligence, Vol. 33, 7370–7377.

[B148] Yin W. Kann K. Yu M. Schütze H. (2017). Comparative study of cnn and rnn for natural language processing. arXiv [Preprint]. arXiv:1702.01923.

[B149] Yoshua B. (2009). Learning deep architectures for AI. Foundat. Trends Mach. Learn. 2, 1–127. 10.1561/2200000006

[B150] Young T. Hazarika D. Poria S. Cambria E. (2018). Recent trends in deep learning based natural language processing. IEEE Comput. Intell. Mag. 13, 55–75. 10.1109/MCI.2018.2840738

[B151] Yu D. Li J. (2017). Recent progresses in deep learning based acoustic models. IEEE/CAA J. Autom. Sinica 4, 396–409. 10.1109/JAS.2017.7510508

[B152] Zhang S. Zhou J. Hu H. Gong H. Chen L. Cheng C. (2015). A deep learning framework for modeling structural features of rna-binding protein targets. Nucleic Acids Res. 43:e32 10.1093/nar/gkv1025 26467480 PMC4770198

[B153] Zhang X. Zhao J. LeCun Y. (2015). Character-level convolutional networks for text classification, in Advances in Neural Information Processing Systems, 649–657.

[B154] Zhou Y. (2019). Sentiment classification with deep neural networks (Master's thesis). Tampere University, Tampere, Finland.

